# A circular RNA activated by TGFβ promotes tumor metastasis through enhancing IGF2BP3-mediated *PDPN* mRNA stability

**DOI:** 10.1038/s41467-023-42571-1

**Published:** 2023-10-28

**Authors:** Ke Li, Jiawei Guo, Yue Ming, Shuang Chen, Tingting Zhang, Hulin Ma, Xin Fu, Jin Wang, Wenrong Liu, Yong Peng

**Affiliations:** 1 Laboratory of Molecular Oncology, Frontiers Science Center for Disease-related Molecular Network, State Key Laboratory of Biotherapy and Cancer Center, West China Hospital, Sichuan University, Chengdu, China; 2 Frontier Medical Center, Tianfu Jincheng Laboratory, 610212 Chengdu, China

**Keywords:** Non-coding RNAs, Metastasis, Epithelial-mesenchymal transition

## Abstract

Metastasis is the leading cause of cancer-related death, where TGFβ-induced epithelial-mesenchymal transition (EMT) process confers on cancer cells increased metastatic potential. However, the involvement of circRNAs in this process is still obscure. Here, we identify a TGFβ-induced circRNA called circITGB6 as an indispensable factor during the TGFβ-mediated EMT process. circITGB6 is significantly upregulated in metastatic cancer samples and its higher abundance is closely correlated to worse prognosis of colorectal cancer (CRC) patients. Through gain- and loss-of-function assays, circITGB6 is found to potently promote EMT process and tumor metastasis in various models in vitro and in vivo. Mechanistically, circITGB6 enhances the mRNA stability of PDPN, an EMT-promoting gene, by directly interacting with IGF2BP3. Notably, interfering circITGB6 with PEI-coated specific siRNA effectively represses liver metastasis. Therefore, our study reveals the function of a TGFβ-regulated circRNA in tumor metastasis and suggests that targeting circITGB6 is a promising strategy for cancer therapy.

## Abstract (web summary)

TGFbeta is known to promote cancer metastasis through the promotion of epithelial-mesenchymal transition (EMT). Here, the authors demonstrate that a TGFbeta induced circular RNA (circITGB6) promotes colorectal cancer metastasis via activation of the m6A reader protein, IGF2BP3, resulting in stabilization of an EMT-promoting gene, PDPN.

## Introduction

Tumor metastasis is the primary cause of mortality, accounting for about 90% of cancer deaths^1^. During the early stage of metastasis, a subset of cancer cells undergoes epithelial-mesenchymal transition (EMT) by which they acquire mesenchymal features and were thus equipped with the ability to disseminate from their primary sites and travel to distant organs^2^. Once cancer cells escape from the primary sites, the cancer would develop an incurable disease as the metastatic cancer cells are hardly eradicated^3^. Therefore, it is urgent to elucidate the mechanisms underlying the metastatic process and identify feasible therapeutic targets to overcome tumor metastasis.

As a critical mediator of cancer metastasis, transforming growth factor beta (TGFβ) signaling pathway is activated by specific interaction of TGFβ with its membrane receptors. Activated TGFβ pathway equips cancer cells with increased migration capability by inducing EMT process, where it decreases the expression of epithelial markers such as E-cadherin and increases the expression of mesenchymal markers like N-cadherin and vimentin^4,5^. Apart from these EMT markers, podoplanin (PDPN), a glycosylated transmembrane protein, also serves as a critical functional effector during EMT process by promoting tumor metastasis^6,7^. Moreover, high *PDPN* expression was reported to be associated with shortened survival of different cancer patients^8–11^, Despite that PDPN can be induced upon TGFβ stimulation^12,13^, the detailed crosstalk between TGFβ and PDPN has not been extensively elucidated.

Increasing evidence supports a role of dysregulated circular RNAs (circRNAs) in tumor metastasis^14–16^. As a class of covalently closed RNA molecules, circRNAs are produced by back-splicing of primary transcripts, which are elaborately controlled under physiological conidiations^17,18^. However, abnormal splicing of pre-mRNAs under certain stress may cause dysregulation of circRNA biogenesis. For example, TGFβ stimulation changed expression of a subset of circRNAs by activating the splicing factor Quaking (QKI) to regulate circRNA biogenesis^19^. As a consequence, dysregulated circRNAs affect the hallmarks of cancer by acting as miRNA sponges, protein decoys or the template to encode small peptides^20,21^. Although the association between circRNAs and tumor metastasis has recently been documented^14–16^, the detailed implication of circRNAs in TGFβ-mediated EMT process remains elusive. Dissection on this issue is of paramount importance, considering the unsatisfactory performance of TGFβ inhibitors as anti-metastatic agents under clinical evaluation^22^.

In this study, we identify that a highly conserved circRNA derived from *ITGB6*, circITGB6 (hsa_circ_0056856), is robustly induced by TGFβ and significantly upregulated in human metastatic cancer samples. Besides, circITGB6 potently elicits EMT process and promotes in vivo metastasis of multiple cancers. Moreover, the metastasis-promoting effect of circITGB6 is largely attributed to its capacity of directly interacting with IGF2BP3 to stabilize *PDPN* mRNA, which encodes a critical effector protein to induce EMT and metastasis. Importantly, in vivo delivery of PEI-coated circITGB6 siRNA complex dramatically suppresses liver metastasis and extends survival of mice suffering liver metastasis. Our findings indicate circITGB6 as a pivotal TGFβ effector and a potential therapeutic target for cancer therapy.

## Results

### circITGB6 is significantly upregulated during TGFβ-induced EMT

**Fig. 1:**
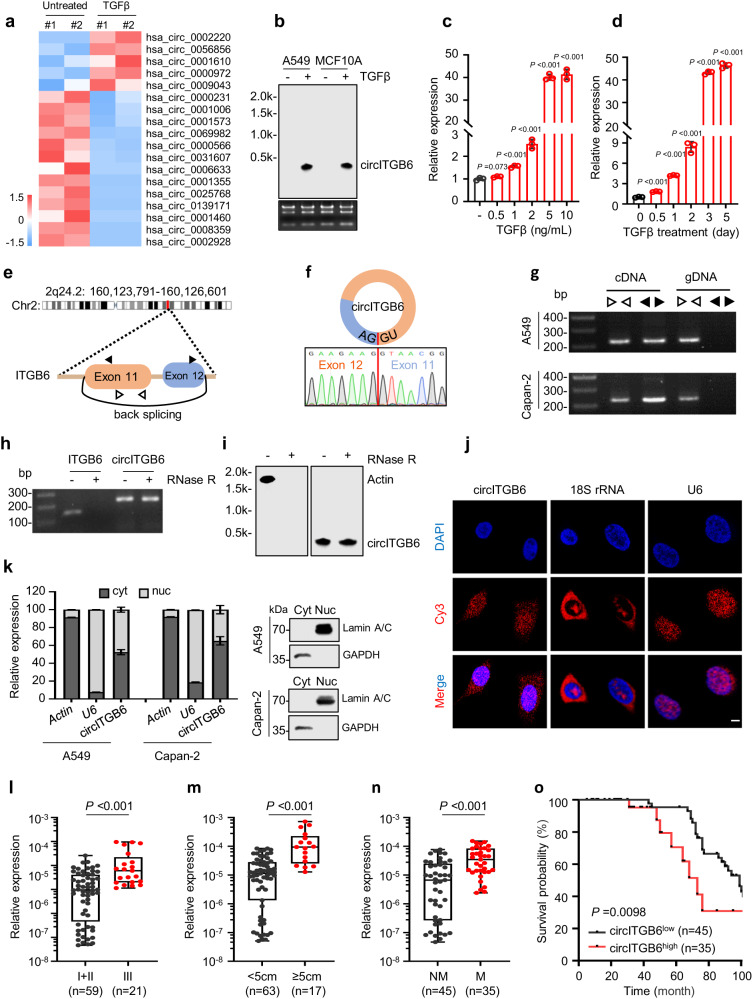
Characterization of the TGFβ-induced circRNA circITGB6. **a** Heat map of TGFβ-responsive circRNAs (annotated in circBase) with high abundance (average RPM > 0.1) and significance (*P* < 0.05). **b** Northern blotting assays for circITGB6 expression in MCF10A and A549 cells with or without 5 ng/ml TGFβ treatment for 72 h. The probe used for Northern blotting crosses the back-splicing junction site of circITGB6. **c**, **d** TGFβ increased cir**c**ITGB6 expression in both time-dependent and dose-dependent manners. A549 cells were untreated (-) or treated with the indicated dose of TGFβ for 72 h (**c**) or with 5 ng/ml TGFβ for the indicated time (**d**). **e** The genomic loci of circITGB6. **f** Sanger sequencing validation of head-to-tail splicing (indicated by red line) between exon 11 and exon 12 of ITGB6 gene. **g** RT-PCR assays were performed from cDNA or gDNA to detect *ITGB6* mRNA or circITGB6 using divergent and convergent primers (indicated by black and white triangle in (**e**)), respectively. **h**, **i** RT-PCR (**h**) and Nort**h**ern blotting (**i**) assays of circITGB6, *ITGB6* or *Actin* mRNA. Total RNAs from A549 cells were digested with or without RNase R. **k** Subcellular distribution of circITGB6 determined by qPCR assays. The cytoplasmic/nucleus fractionations were prepared in A549 and Capan-2 cells. *Actin* and *U6* transcripts were used as the cytoplasmic and nucleus marker, respectively. Immunoblot assays with anti-GAPDH and anti-Lamin A/C confirmed good cytoplasmic/nucleus fractionation. Cyt cytoplasmic; Nuc nuclear. **j** RNA fluorescence in situ hybridization for circITGB6. 18 S rRNA and U6 were used as the cytoplasmic and nuclear marker, respectively. Nuclei were stained with DAPI. Scale bar, 10 µm. **l–n** qPCR assays for exploring the clinical relevance of circITGB6 expression in human CRC cancer tissues (*n* = 80 samples, cohort 1). Cancer grade (**l**), tumor size (**m**) and lymph node metastasis status (**n**). The horizontal lines represent the median; the bottom and top of the boxes represent the 25th and 75th percentiles, respectively; and the vertical bars represent the range of the data. **o** Kaplan–Meier curve for the overall survival of CRC patients, which were divided into two groups according to the mean expression of circITGB6 (*n* = 80 samples, cohort 1). Data represent mean ± sd from three independent experiments (**c**, **d**). The *P* value was calculated by unpaired, two-tailed Student’s *t* test (**a**, **c**, **d**), one-way ANOVA test (**l–n**) and two-sided log-rank test (**o**). Source data are pr**o**vided as a Source Data file.

Considering that normal breast epithelial MCF10A cells is well-established cell model for exploring the TGFβ-induced EMT process and cancer progression, MCF10A cells were treated with or without TGFβ and then subjected to bioinformatics analysis of their transcriptome profiles to identify TGFβ-regulated circRNAs (GEO accession number: GSE165576). Among the TGFβ-upregulated circRNAs, hsa_circ_0056856 showed the most striking increase in the TGFβ-treated group (Fold change = 38.57, *P* value = 0.0218) (Fig. 1a). To verify these findings, we stressed epithelial MCF10A and A549 cells with TGFβ (Supplementary Fig. 1a), then detected circRNA levels by qPCR experiments. The results indicated circITGB6 as the most strikingly increased one (Supplementary Fig. 1b, c and Supplementary Table 1). Besides, TGFβ-induced circITGB6 expression was validated by Northern blotting assays, using biotin-labeled probes crossing the junction site (Fig. 1b). Moreover, TGFβ increased circITGB6 expression at a time- and dose-dependent manner (Fig. 1c, d). Notably, we also expanded our findings in CRC cell models where HCT116 and HT29 cells undergoing a typical EMT upon TGFβ exhibited striking increase of circITGB6 (Supplementary Fig. 1d–f and Supplementary Table 1). Collectively, these results supported circITGB6 as a TGFβ-induced circRNA in multiple cell models.

### Characteristics of circITGB6

Our bioinformatics analysis indicated that circITGB6 arises from exons 11 to 12 of the *ITGB6* gene with 321 nucleotides in length (Fig. 1e), which was experimentally confirmed by RT-PCR using divergent primers and Sanger sequencing (Fig. 1f), consistent with the annotation in circBase (http://www.circbase.org/). Moreover, circITGB6 was only amplified with divergent primers using cDNAs, rather than genomic DNAs, as the templates (Fig.1g), thus excluding that circITGB6 was formed from genomic rearrangements or PCR artifacts. Compared to the linear transcripts of *ACTIN* and its host gene *ITGB6*, circITGB6 was resistant to digestion of the RNA exonuclease RNase R (Fig. 1h, i), further supporting that circITGB6 is a circular RNA. To assess the subcellular distribution of circITGB6, cytoplasmic and nuclear RNAs were extracted for qPCR analysis. As shown in Fig. 1k, circITGB6 localized in both cytoplasm and nuclei in A549 and Capan-2 cells, and further confirmed by FISH assays in A549 cells (Fig. 1j). Next, we sought to explore the mechanism that TGFβ increased circITGB6 expression. Recently, Conn et al. reported that QKI-mediated back-splicing was involved in TGFβ-induced EMT process^19^. To check whether QKI is involved in circITGB6 biogenesis, qPCR assays were performed in OKI overexpression (OE) or knockdown (KD) A549 cells and the results showed that QKI barely affected circITGB6 abundance (Supplementary Fig. 1h, i), indicating that other mechanism may contribute to the circITGB6 upregulation upon TGFβ stimulation. Given that increased transcription of host gene may upregulate circRNA expression, we measured the expression of circITGB6 host transcript, *ITGB6* mRNA, upon TGFβ stress. ITGB6 is an important integrin subunit for TGFβ signaling pathway and cancer metastasis^23^. As expected, TGFβ obviously increased ITGB6 expression (Supplementary Fig. 1g). In accordance, pre-mRNA levels of *ITGB6* als-ed upon TGFβ stimulation (Supplementary Fig. 1j), implying that the increased circITGB6 may be attributed to activated transcription of its host gene. Notably, Northern blotting data showed a similar increase of both circITGB6 and *ITGB6* under TGFβ treatment, using probes targeting their shared exons 11/12 (Supplementary Fig. 1k). Intriguingly, quantification data indicated circITGB6 as the dominate TGFβ-induced product, compared to its host transcript (Supplementary Fig. 1l).

To explore whether circITGB6 is conserved among species, we predicted a mouse homologous variants (circItgb6) derived from the exon 11/12 of *Itgb6* gene through bioinformatics analysis (Supplementary Fig. 2a, b). As expected, RT-PCR assays produced the predicted bands using different divergent primers from mouse cell lines or tumor tissues (Supplementary Fig. 2c, d), and the back-splice junction site of circItgb6 was verified by Sanger sequencing (Supplementary Fig. 2e). Moreover, the sequence identity of human circITGB6 and mouse circItgb6 was 84.24% (Supplementary Fig. 2f), demonstrating that circITGB6 is highly conserved between human and mouse. Intriguingly, circItgb6 was not detected in normal mouse breast tissues, but highly expressed in cancerous tissues (Supplementary Fig. 2c), implying its oncogenic role during tumorigenesis. To check whether mouse circItgb6 was also induced by TGFβ in mouse system, we used an epithelial murine breast cancer cell line Py2T^24^, which underwent a complete EMT upon treatment with TGFβ (Supplementary Fig. 2g, h). As expected, mouse circItgb6 was also robustly induced by TGFβ (Supplementary Fig. 2i). Collectively, circITGB6 is highly conserved in human and mouse.

### Higher circITGB6 expression is associated with poorer outcomes of CRC patients

TGFβ plays a critical role in tumor metastasis^25^, the implication of circITGB6 in TGFβ signaling (Fig. 1a–d and Supplementary Figs. 1a–f, 2i) as well as tumorigenesis (Supplementary Fig. 2c) prompted us to investigate the correlation between circITGB6 and tumor metastasis. To this end, a cohort of 80 CRC samples was employed to detect circITGB6 abundance. A significant increase of circITGB6 expression was observed in the high stage (stage III), large-size tumor (≥5 cm) as well as CRC with lymph node metastasis (cohort 1, Fig. 1l–n and Supplementary Table 2). Given that metastasis is a leading cause of cancer dearth^26^, we investigated the correlation between circITGB6 expression and patient survival in the same CRC samples. As expected, higher circITGB6 level is remarkably associated with worse prognosis of individuals with CRC (Fig. 1o, Supplementary Table 3). Thus, our results indicated that circITGB6 expression may be a potential predictor for both metastasis and prognosis of CRC patients.

### circITGB6 elicits EMT process and promotes tumor metastasis

**Fig. 2:**
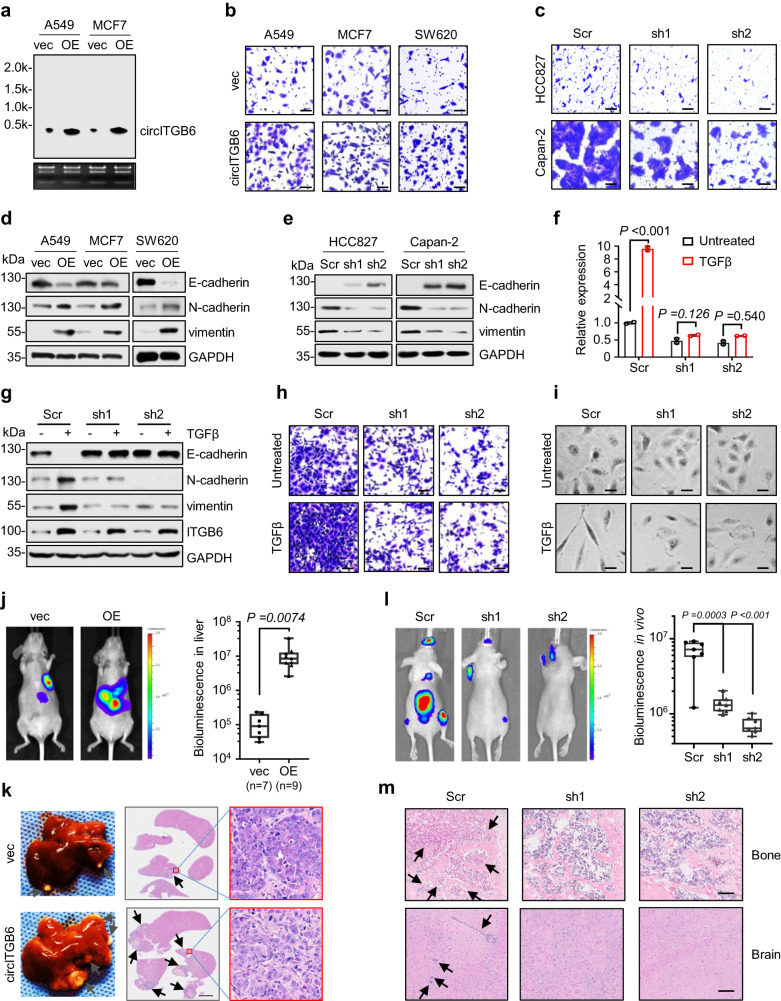
circITGB6 facilitated EMT process and tumor metastasis. **a** Northern blotting assays for circITGB6 expression in A549 and MCF7 cells with circITGB6 overexpression. **b**, **c** Transwell migration assays for circITGB6 overexpression (**b**) and knockdown (**c**) cells. Scale bar, 20 μm. Vec, vector; Scr, scramble shRNA; sh1 and sh2 represent two independent shRNAs targeting the back-splicing region of circITGB6. **d**, **e** Effects of circITGB6 overexpression (**d**) and knockdown (**e**) on the protein levels of EMT markers. GAPDH as loading control. **f–i** qPCR assays for circITGB6 expression (**f**), immunoblotting assay for the expression of EMT markers and ITGB6 (**g**), Transwell migration assay (**h**) and representative images of cell morphology (**i**) in A549 stable cells with or without circITGB6 knockdown after 5 ng/mL TGFβ treatment for 72 h. **j**, **k** Stably expressing vector or circITGB6 SW620 cells (1 × 10^6^) were injected into the spleen to generate liver metastases. **j** Representative bioluminescence images of liver metastasis and quantitation of bioluminescence were presented. **k** Representative images for H&E staining of mouse liver lesions. The metastatic nodules indicated by the black arrows. Scale bar, 4 mm. *n* = 7 mice (vec) or 9 (OE) mice per group (**j**, **k**). **l**, **m** Capan-2 cells (1 × 10^5^) stably expressing scramble or circITGB6 shRNAs (sh1 and sh2) were intracardially injected for metastasis assays, *n* = 7 mice (Scr, sh1 or sh2) per group. Representative bioluminescence imaging of tumor metastasis and quantitation of bioluminescence were presented (**l**). Representative images for H&E staining of mouse brains or right femur tissues (**m**). The metastatic nodules indicated by the black arrows. Scale bar: 100 μm. The horizontal lines represent the median; the bottom and top of the boxes represent the 25th and 75th percentiles, respectively; and the vertical bars represent the range of the data (**j**, **i**). Data represent mean ± sd from three independent experiments (**f**), mean ± s.e.m (**j**, **i**). Significance of differences was determined by one-way ANOVA test (**j**, **l**) and two-tailed Student’s *t* test (**f**). Source data are provided as a Source Data file.

After documenting the characteristic of circITGB6 and its correlation to tumor metastasis, we sought to investigate its biological function in in vitro and in vivo models. To this end, we generated circITGB6-OE and -KD cell lines, as verified by Northern blotting assays (Fig. 2a), RT-PCR or qPCR assays (Supplementary Fig. 3a, e, f). Besides, the exogenously expressed circITGB6 demonstrated same subcellular distribution as its endogenous counterpart (Supplementary Fig. 3b, c). Furthermore, neither circITGB6 OE nor KD affected the expression of its host gene ITGB6 (Supplementary Fig. 3d–f), thus excluding that circITGB6 plays its biological functions through ITGB6 pathway.

In accordance to the aforementioned implication of circITGB6 in TGFβ signaling, circITGB6 overexpression significantly promoted cell migration in lung cancer A549, breast cancer MCF7 and CRC SW620 cells (Fig. 2b), while knockdown of circITGB6 dramatically weakened cell migration in lung cancer HCC827 and pancreatic cancer Capan-2 cells (Fig. 2c), with limited effect on cell proliferation (Supplementary Fig. 3g, h). Besides, circITGB6 overexpression decreased the epithelial marker E-cadherin level, while increased the mesenchymal makers N-cadherin and vimentin levels in A549, MCF7 and SW620 cells (Fig. 2d). In accordance, circITGB6 knockdown re-shaped the expression of these EMT markers in HCC827 and Capan-2 cells (Fig. 2e), which further confirmed in HCT116 and SW480 cells (Supplementary 3i, j). These results collectively support a promoting role of circITGB6 in cell migration and EMT process.

To confirm the dependency of TGFβ signaling activation on circITGB6, we treated A549 cells with TGFβ after stable knockdown of the endogenous circITGB6 using two independent circITGB6-specific shRNAs. qPCR analysis revealed sustained and efficient knockdown of circITGB6 expression under TGFβ stress (Fig. 2f). As shown in Fig. 2g, circITGB6 depletion dramatically blocked the upregulation of N-cadherin and vimentin as well as the downregulation of E-cadherin triggered by TGFβ stimulation. In accordance, such EMT-antagonistic effects of circITGB6 knockdown were also observed on TGFβ-induced cell migration (Fig. 2h) and morphological transition (Fig. 2i). Collectively, these results supported a potent permissive effect of circITGB6 on TGFβ-induced EMT process.

To explore the role of circITGB6 on tumor metastasis in vivo, we first performed splenic injection of SW620 cells with or without stable circITGB6 overexpression in immunodeficient nude mice to generate a liver metastasis model. circITGB6-overexpressed SW620 cells seemed to be more liable to form metastatic lesions in mouse livers when compared to the control cells, as evidenced by increased bioluminescent signals and more H& E stained tumors in the livers (Fig. 2j, k). To further substantiate these findings, we next developed systemic metastasis model based on intracardiac injection to mimic clinical parameters of cancer progression. circITGB6 knockdown significantly decreased the number and size of metastatic lesions in brain and bone (Fig. 2l, m). In addition, in tail-vein injection mouse model, circITGB6 overexpression resulted in a substantial increase in the number and size of lung metastases (Supplementary Fig. 3k, l). All these data indicate that circITGB6 potently promotes tumor metastasis in vivo.

TGFβ may induce EMT process through ncRNAs like miR-200 or lncRNA-ATB and the core EMT-related transcriptional factors (TFs)^27,28^. To explore the association of circITGB6 with the previously reported pathways, qPCR and immunoblotting assays were performed. Intriguingly, circITGB6 barely affected the levels of lncRNA-ATB and miR-200, nor the expression of core EMT-related TFs (Supplementary Fig. 4a, b). Moreover, overexpression of these EMT-related TFs in A549 cells alone minimally increased circITGB6 expression (Supplementary Fig. 4c, d). Notably, circITGB6 could also promote cell migration after depleting its host gene *ITGB6* (Supplementary Fig. 4e–g). Also, circITGB6 also had no effect on the expression of TGFβ downstream factors (Supplementary Fig. 4h). These results collectively support that circITGB6 facilitates TGFβ-induced EMT process and tumor metastasis with limited correlation with the reported signaling pathways.

### circITGB6, rather than its linear transcript, plays the metastasis-promoting effect

**Fig. 3:**
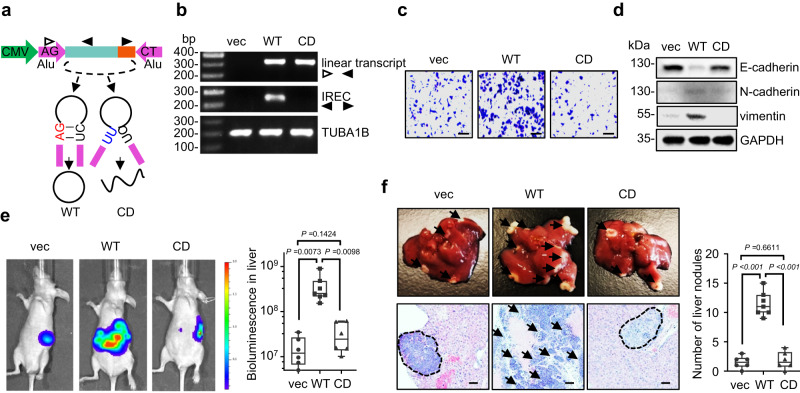
The metastatic role of circITGB6 is independent of its linear transcripts. **a** Schematic representation of circularization-dead (CD) circITGB6 plasmids. CMV, cytomegalovirus promoter; Alu, Alu sequences. **b** RT-PCR assays showed the failure of exogenous circITGB6 formation in circITGB6-CD SW620 stable cells. **c**, **d** Transwell migration assays (**c**) and Immunoblotting assays of EMT markers (**d**) in circITGB6 wild-type (WT) or CD stable cells. GAPDH was used as the loading control. Scale bar: 20 μm. **e** Bioluminescence imaging of liver metastasis and quantitation of bioluminescence of vec, circITGB6-WT and -CD groups. *n* = 6 mice (vec or CD) or 7 mice (WT) per group. **f** Representative images of liver metastatic nodules and H&E staining of mouse liver tissues (*left*) and the numbers of liver metastatic nodules (*right*). The metastatic nodules indicated by the black arrows or dotted lines. Scale bar: 20 μm. The horizontal lines represent the median; the bottom and top of the boxes represent the 25th and 75th percentiles, respectively; and the vertical bars represent the range of the data (**e**, **f**). Data represent mean ± s.e.m (**e**, **f**). Significance of differences was determined by one-way ANOVA test (**e**, **f**). Source data are provided as a Source Data file.

Considering that the circRNA-overexpressing plasmid produces large amounts of linear transcripts that are not completely circularized into circular RNAs^29^, the observed phenomena could be caused by linear transcripts. To rule out this possibility, we mutated the circITGB6-OE plasmid from AG to TT within the circularization-assisting elements to generate the circulation-dead (CD) mutant (Fig. 3a). As shown in Fig. 3b, circITGB6-CD plasmids efficiently expressed linear transcripts, but cannot form exogenous circITGB6 in SW620 cells. As expected, compared to its wild-type counterpart, circITGB6-CD had no obvious effects on expression of EMT makers (Fig. 3c) and cell migration (Fig. 3d). Next, we further confirmed the above findings in a liver metastasis model by intra-splenic injections of the aforementioned SW620 cells. The results showed that mice injected with circITGB6-CD cells exhibited comparable metastatic lesions to those bearing empty vector cells, whereas mice bearing circITGB6-WT cells have more severe liver metastasis (Fig. 3e, f). The failure of circITGB6-CD to promote liver metastasis exclude that the metastatic role of circITGB6 is attributed to its linear transcripts. Therefore, circITGB6 elicited EMT process and tumor metastasis independent of its linear transcripts.

### circITGB6 interacts with IGF2BP3 to promote tumor metastasis

Increasing evidence indicates that circRNAs participate in different biological processes through distinct mechanisms, such as miRNA sponge^30,31^, protein interaction^15,32^, or translating into functional peptides^33–35^. Our bioinformatics analysis using TransCirc^36^ and riboCIRC^37^ database showed that circITGB6 had limited potential to encode peptides due to the lack of putative IRES element, or to act as miRNAs sponge because circITGB6 has no more than two potential binding-sites for any miRNAs, so circITGB6 may function as a protein decoy.

**Fig. 4:**
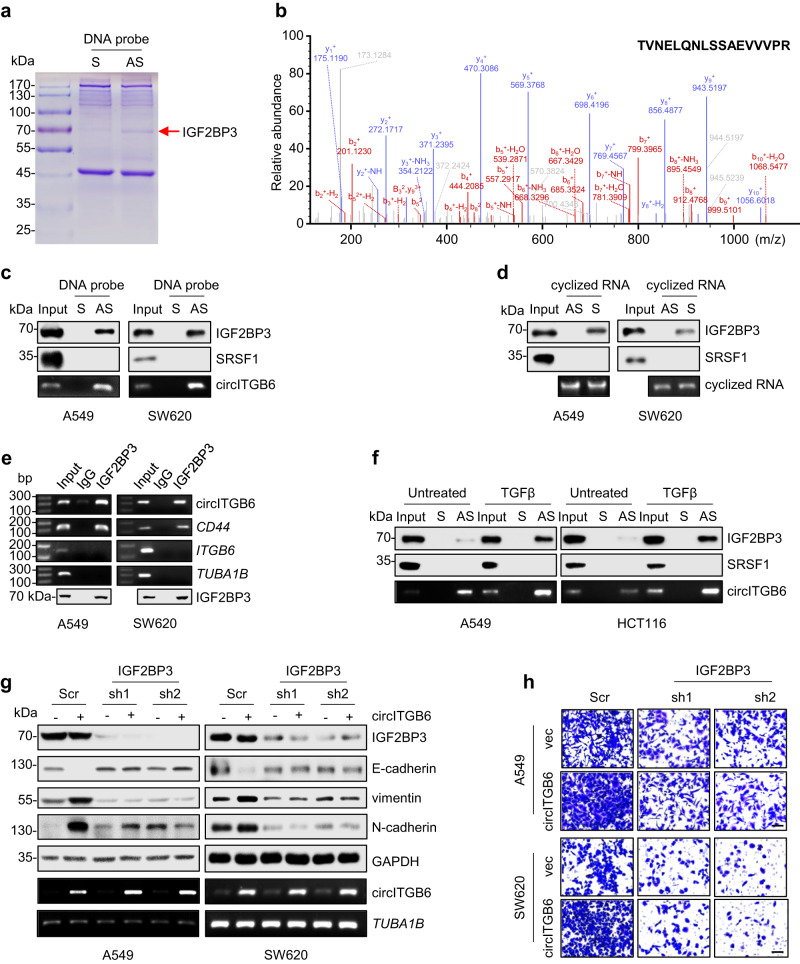
circITGB6 interacts with IGF2BP3 to promote tumor metastasis. **a** Coomassie blue staining of circITGB6-interacting proteins pulled down by the antisense (AS) DNA probes from A549 cell lysate. (S) and AS represent the DNA probes identical and complementary to circITGB6 sequences crossing back-splicing junction site, respectively. The band shown by red arrow was subjected to mass spectrometry. **b** Representative IGF2BP3 peptide detected by mass spectrometry in circITGB6 pull-down fractions. **c**, **d** Immunoblot assays of IGF2BP3 pulled down by DNA probes (**c**) or in vitro transcribed and circularized circITGB6 (**d**) from A549 or SW620 cell lysates. SRSF1 served as a negative control. Enrichment efficiency was determined by detecting circITGB6 abundance in the precipitation (**c**). **e** RIP assays showed the association of IGF2BP3 with circITGB6 in A549 or SW620 cells using anti-IGF2BP3 antibody or the control IgG. *CD44* served as the positive control. *TUBA1B* served as the negative control. **f** Immunoblotting assays of IGF2BP3 pulled down by DNA probes in A549 or HCT116 cells with or without TGFβ treatment. SRSF1 served as the negative control. Enrichment efficiency was determined by detection of circITGB6 abundance in the precipitation. **g**, **h** Immunoblot assays of EMT markers (**g**) and Transwell migration assays (**h**) for circITGB6 overexpression A549 or SW620 stable cells with or without IGF2BP3 knockdown. Scale bar: 20 μm. Source data are provided as a Source Data file.

To identify the circITGB6-interacting proteins, we performed RNA pull-down assays after introducing biotinylated sense (S) or antisense (AS) DNA oligomers crossing circITGB6 back-splicing junction site into cells, then the circITGB6-bound proteins were subjected to SDS-PAGE and Coomassie blue staining. As shown in Fig. 4a, a protein band with ~70 kDa (red arrow) was specifically pulled down by the AS probe and excised for mass spectrometry analysis. An RNA-binding protein, insulin like growth factor 2 mRNA binding protein 3 (IGF2BP3), was identified with the top abundance (unique peptide = 25, coverage = 48%) and highest scores (Score Sequest HT = 346.9) among the potential interacting proteins of circITGB6 (Fig. 4b and Supplementary Table 4). The association of circITGB6 with IGF2BP3 was validated using antisense probes (Fig. 4c) or the in vitro transcribed and circularized circITGB6 (Fig. 4d). Besides, RNA immunoprecipitation (RIP) assays using anti-IGF2BP3 antibody further confirmed the interaction between IGF2BP3 and circITGB6 (Fig. 4e), where *CD44* mRNAs serve as a positive control^38^. Notably, the linear transcripts of *ITGB6*, the host gene of circITGB6, showed minimal binding to IGF2BP3 (Fig. 4e), indicating that IGF2BP3 specifically bound to circITGB6, rather than its host gene transcript. Moreover, endogenous IGF2BP3-circITGB6 association was strengthened under TGFβ stimulation, as a consequence of increased circITGB6 abundance (Fig. 4f). By performing immunofluorescence and fluorescence in situ hybridization (IF-FISH) assays, we confirmed the cytoplasmic colocalization of endogenous circITGB6 and IGF2BP3 in A549 cells, without being affected by circITGB6 knockdown or TGFβ stress (Supplementary Fig. 5). Thus, our results strongly support that the interaction between circITGB6 and IGF2BP3 is not cell-type/tissue-specific. To address whether IGF2BP3 contributes to the metastasis-promoting effect of circITGB6, two independent shRNAs targeting IGF2BP3 were generated and delivered into circITGB6-OE stable cells. The results showed that knockdown of IGF2BP3 weakened circITGB6 effects on the expression of EMT markers (Fig. 4g) and cell migration (Fig. 4h). Collectively, IGF2BP3 may function as a critical circITGB6-associating factor to promote EMT process and cell migration.

### The ‘CAUU’ region within circITGB6 is crucial for its direct interaction with IGF2BP3

**Fig. 5:**
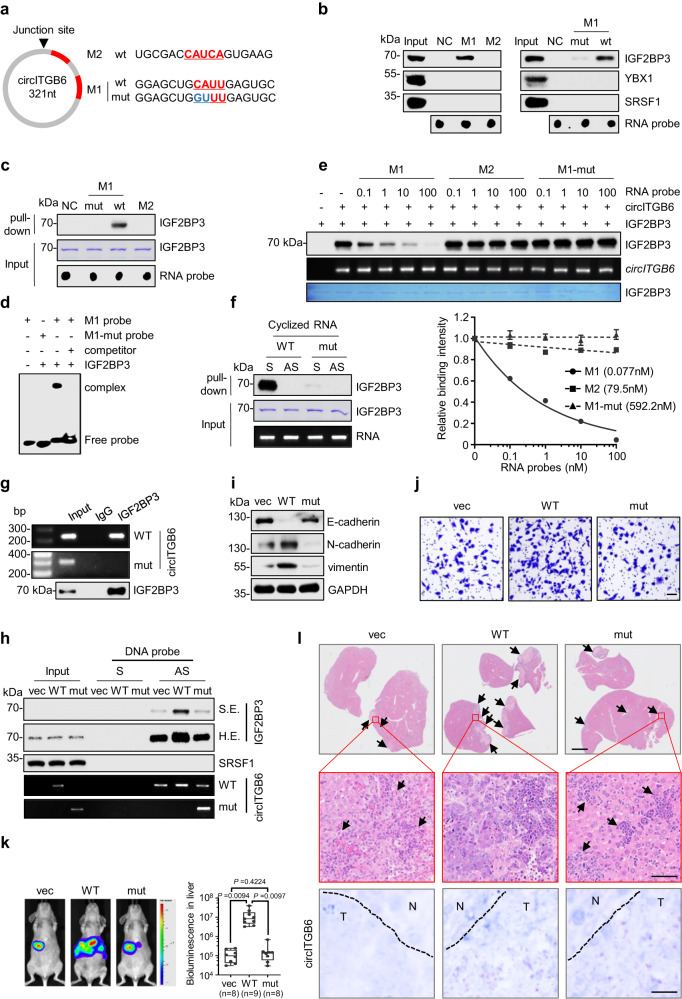
Identification of the IGF2BP3-binding site within circITGB6 sequence. **a** Schematic representation of two putative IGF2BP3-binding regions within circITGB6 that contain stretches of CAUH (H = A, C, or U) element (colored in red). The 3′-end biotin-labeled probes (M1-WT, M1-mut (mutated nucleotides shown in blue) and M2) were synthesized. **b** RNA pull-down assays using different RNA probes with A549 cell lysates. The amounts of probes were monitored by the HRP-streptavidin. **c**, **d** In vitro RNA pull-down assays (**c**) and RNA-EMSA assays (**d**) showing the direct binding of purified recombinant IGF2BP3 protein with biotin-labeled M1 probe. **e** (*Upper*) in vitro RNA pull-down assays showing the enrichment of IGF2BP3 by biotin-labeled circITGB6 in the presence of non-biotin probes (NC, M1, M2 or M1-mut). (*bottom*) Relative binding intensity derived from signal quantification of three independent experiments (mean ± sd), Band intensities were normalized to control NC samples for each experiment (set at a value of 1.0). **f** RNA pull-down assays using the purified recombinant IGF2BP3 protein and in vitro circularized wild-type (WT) or mutant circITGB6. **g** RIP assays were performed with anti-IGF2BP3 antibody or the normal IgG in A549 cells stably expressing circITGB6 WT or IGF2BP3-interaction deficiency mutant (mut), and RT-PCR assays for the enrichment of WT or mutant circITGB6. **h** RNA pull-down assays from different A549 stable cells using DNA probes. S and AS represent the DNA probe identical and complementary to circITGB6 sequence crossing back-splicing junction site, respectively. **i**, **j** Immunoblot assays of EMT markers (**i**) and Transwell migration assays (**j**) in SW620 cells stably expressing circITGB6 WT or mutant. **k** Representative bioluminescence images and bioluminescence quantitation of tumor metastasis in liver. *n* = 8 mice (vec or mut) or 9 mice (WT) per group. The horizontal lines represent the median; the bottom and top of the boxes represent the 25th and 75th percentiles, respectively; and the vertical bars represent the range of the data. Data represent mean ± s.e.m. Significance of differences was determined by one-way ANOVA test. **l** Representative images for H&E staining and the foci of circITGB6 (red point) of mouse liver tissues. The metastatic nodules indicated by the black arrows. Scale bar: 20 μm. Source data are provided as a Source Data file.

Markus et al. reported that IGF2BP3 preferentially bound to the “CAUH (H = A, U or C)” consensus element^39^. To elucidate the molecular basis of the association between circITGB6 and IGF2BP3, utilizing RBPmap^40^ database, we predicted two potential IGF2BP3-binding regions (containing either “CAUU” or “CAUCA”) within circITGB6 and generated two biotin-labeled RNA probes M1 and M2 (Fig. 5a). RNA pull-down assays revealed that endogenous IGF2BP3 did not bind to M2 probe, but preferentially to M1 probe (Fig. 5b, left). To figure out whether the CAUU motif contributes to IGF2BP3 association, an M1 mutant (M1-mut) RNA probe was generated by double-nucleotide replacement of “CAUU” to “GUUU” (Fig. 5a). The results showed that M1-mut remarkably abolished the interaction between IGF2BP3 and circITGB6 (Fig. 5b, right), thus supporting a critical role of this ‘CAUU’ motif in mediating circITGB6-IGF2BP3 association. Next, to confirm the direct interaction of IGF2BP3 with M1 sequence, the recombinant IGF2BP3 protein was incubated with the aforementioned RNA probes. The direct interaction between IGF2BP3 and M1 was observed in both RNA pull-down (Fig. 5c) and RNA-EMSA assays (Fig. 5d). Moreover, competitive binding assays showed that M1 probe, rather than M2 or M1-mut probes, efficiently displaced IGF2BP3 from biotin full-length circITGB6 (Fig. 5e). These data suggested that IGF2BP3 bound circITGB6 through its CAUU motif. We next wondered whether ‘CAUU’ motif was indispensable for the interaction between IGF2BP3 and circITGB6. To this end, biotin-labeled circITGB6 or its GUUU mutant variant, named circITGB6-mut, were generated by in vitro transcription and circularization, and then incubated with recombinant IGF2BP3 protein. As expected, circITGB6-mut hardly captured IGF2BP3, whereas circITGB6-WT demonstrated strong interaction with IGF2BP3 (Fig. 5f). Furthermore, the endogenous interaction between IGF2BP3 and circITGB6-WT, rather than circITGB6-mut, was validated by both RIP (Fig. 5g) and pull-down assays (Fig. 5h), arguing that circITGB6 directly interacts with IGF2BP3 via the “CAUU” region.

To figure out whether direct interaction of circITGB6 with IGF2BP3 contributes to the metastatic effect of circITGB6, A549 and SW620 cells with enforced expression of circITGB6-WT or equivalent circITGB6-mut variants were employed to evaluate their cell migration and tumor metastasis abilities (Supplementary Fig. 6a). As expected, enforced circITGB6-mut expression failed to promote EMT process according to the findings on EMT marker expressions and cell migration in SW620 cells (Fig. 5i, j) and A549 (Supplementary Fig. 6b, c). Next, we further confirmed the above findings in a liver metastasis model. The results showed that mice injected with circITGB6-mut cells exhibited comparable metastatic lesions to controls, whereas mice bearing circITGB6-WT cells have remarkably increased the number and size of liver metastases (Fig. 5k, l). Similar phenomena were also observed in the mouse in-vein metastasis model where wild-type circITGB6, rather than the IGF2BP3 interaction-deficient circITGB6 mutant, promoted tumor metastasis (Supplementary Fig. 6d), increased metastatic nodules in the lungs (Supplementary Fig. 6e) and facilitated EMT process in metastatic nodules (Supplementary Fig. 6f), further supporting the contribution of the interaction between circITGB6 and IGF2BP3 on tumor metastasis.

### circITGB6 regulates PDPN mRNA stability through its interaction with IGF2BP3

IGF2BP3 plays its biological roles by modulating RNA metabolism, mRNA translation and intracellular location^41–43^. Given the facts that circITGB6 promotes EMT process and cell migration through its association with IGF2BP3 (Fig. 4g, h), with limited effect on IGF2BP3 abundance (Figs. 4g, 5h and Supplementary Fig. 5), we wondered if circITGB6 promotes tumor metastasis through modulating certain mRNA cargos of IGF2BP3.

**Fig. 6:**
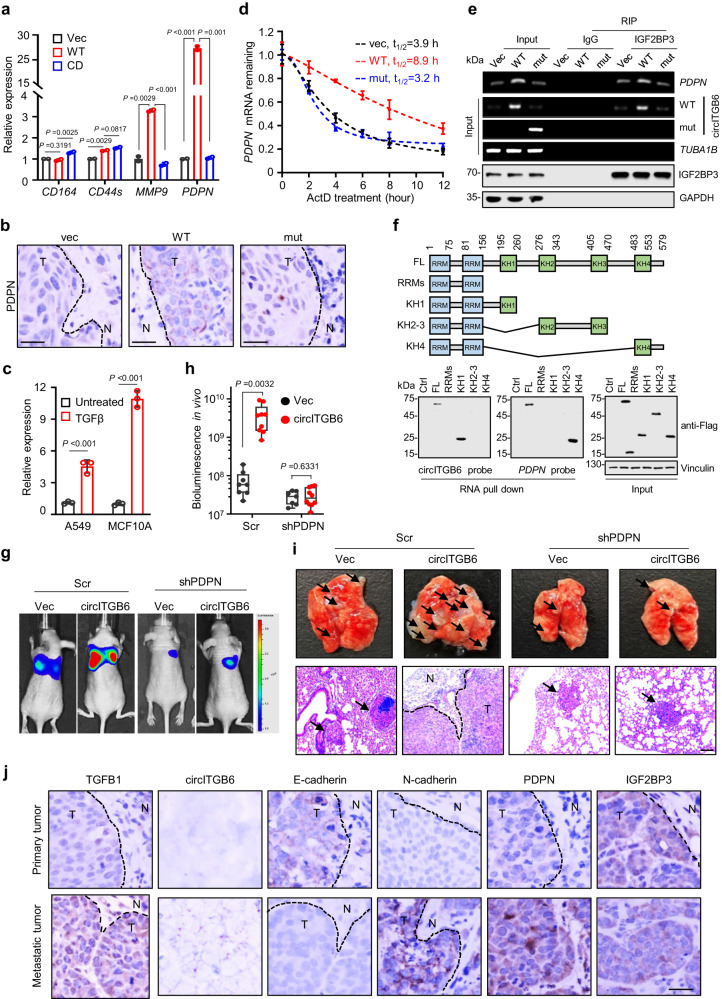
circITGB6 regulates PDPN mRNA stability through its interaction with IGF2BP3. **a** qPCR assays for *CD164*, *CD44s*, *MMP9* and *PDPN* expression in Vec, circITGB6-WT and circITGB6-CD SW620 stable cells. **b** Representative IHC staining images using anti-PDPN antibody of the metastatic lesions in the lung from mice tail-vein injected with A549 stable cells. Scale bar: 20 μm. **c** qPCR assays for *PDPN* mRNA expression in A549 and MCF10A with or without TGFβ treatment. **d** Measurement the half-life of *PDPN* mRNA in Vec, circITGB6-WT and circITGB6–mut A549 cells treated with 5 μM of actinomycin D (ActD) for indicated times. **e** RIP assays with anti-IGF2BP3 or the normal IgG antibody, and the resultant precipitates were subjected to RT-PCR assays. *TUBA1B* and GAPDH served as the negative controls. **f** Top: Schematic diagrams showing RNA-binding domains within IGF2BP3 and the list of different IGF2BP3 truncation mutants. Bottom: Immunoblot assays with anti-Flag antibody after performing RNA pull-down experiments using DNA probes specifically targeting circITGB6 or *PDPN* mRNA in A549 cells transfected with plasmids encoding Flag-tagged full-length (FL) or truncated IGF2BP3 mutants. **g–i** Stably expressing vector or circITGB6 A549 cells (1 × 10^6^) with or without *PDPN* knockdown were tail-vein injected into mice for metastasis assays. *n* = 8 mice per group. Representative bioluminescence images (**g**), bioluminescence quantitation (**h**) and H&E staining images of metastatic lung tissues (**i**) were presented. The metastatic nodules indicated by the black arrows. The horizontal lines represent the median; the bottom and top of the boxes represent the 25th and 75th percentiles, respectively; and the vertical bars represent the range of the data. Data represent mean ± s.e.m. **j** Representative IHC staining images of TGFB1, E-cadherin, N-cadherin, PDPN, IGF2BP3 and the foci of circITGB6 (red point) in the primary within the spleen or metastatic lesions within the liver in mice intra-splenically injected with SW620 cells. Scale bar: 20 μm. Data represent mean ± sd from three independent experiments (**a**, **c**, **d**). Significance of differences was calculated by two-tailed Student’s *t* test (**a**, **c**, **d**) and one-way ANOVA test (**h**). Source data are provided as a Source Data file.

Because Bell et al. summarized that IGF2BP3 can bind to metastasis-related transcripts, such as *CD164*, *CD44s*, *MMP9* and *PDPN* mRNAs^42^, we performed RT-qPCR assays to examine the effects of circITGB6 on their expression. The results showed that enforced expression of circITGB6-WT, rather than its linear transcripts (CD), dramatically increased mRNA levels of MMP9 and PDPN genes in SW620 cells, with *PDPN* showing the most significant increase (Fig. 6a). So, we focused on *PDPN* genes in the following experiments. Consistently, circITGB6 overexpression significantly upregulated *PDPN* mRNA and protein levels (Supplementary Fig. 7a, b). In contrast, circITGB6 knockdown dramatically decreased *PDPN* expression in both CRC HCT116 and SW480 cells (Supplementary Fig. 7c, d). Moreover, the IGF2BP3 interaction-deficient circITGB6 mutant failed to affect *PDPN* expression in A549 cell lines (Supplementary Fig. 7e) and pulmonary metastatic tissues (Fig. 6b). In addition, *PDPN* mRNA level was elevated upon TGFβ stimulation in both A549 and MCF10A cells as a consequence of increased circITGB6 expression (Fig. 6c), consistent with previous findings that TGFβ induced PDPN expression^44,45^. Collectively, our results indicated that *PDPN* may be induced as a consequence of IGF2BP3/circITGB6 interaction.

Hwang et al. reported positive correlation between IGF2BP3 and *PDPN* expression in oral squamous cell carcinoma cells^46^, implying that *PDPN* mRNA may acts as one of the downstream RNA cargos of IGF2BP3. To verify this, we generated stable A549 cells with IGF2BP3 overexpression or knockdown. The results showed that IGF2BP3 positively regulated *PDPN* mRNA and protein levels in lung cancer cells (Supplementary Fig 7f–h). Interestingly, both circITGB6 and IGF2BP3 have little effect on the expression of *PDPN* pre-mRNA in A549 cells (Supplementary Fig. 7i), indicating that circITGB6/IGF2BP3 modulates *PDPN* expression independent on its transcription. Given that IGF2BP3 can stabilize its mRNA cargos^41,42^, we hypothesize that circITGB6 increases *PDPN* mRNA level through its interaction with IGF2BP3 to modulate the half-life of *PDPN* mRNA. To test this, we first measured the half-lives of *PDPN* mRNAs upon enforced expression of different circITGB6 variants. As expected, *PDPN* mRNA stability can be obviously extended by wide-type circITGB6, rather than its IGF2BP3-binding deficient variant (Fig. 6d). Furthermore, enforced expression of circITGB6-WT, rather than circITGB6-mut, significantly increased the enrichment of *PDPN* mRNAs in the IGF2BP3-immunoprecipitated fractions (Fig. 6e). These findings indicate that circITGB6 enhanced the stability of *PDPN* mRNA by promoting the interaction between IGF2BP3 and *PDPN* mRNA.

Considering IGF2BP3 binds to its RNA cargos via different RNA-binding KH domains, we constructed IGF2BP3 truncated mutants with individual KH domains (Fig. 6f, upper) to dissect how IGF2BP3 interacts with circITGB6 and *PDPN* mRNAs, respectively. RNA pull-down assays using probes targeting either circITGB6 or *PDPN* mRNA revealed that KH1 domain of IGF2BP3 specifically bound to circITGB6, while KH4 domain specifically bound to *PDPN* mRNA (Fig. 6f, bottom), excluding that circITGB6 competes with *PDPN* mRNA to bind to the same KH domain of IGF2BP3. RIP assays further confirmed that circITGB6 and *PDPN* mRNA bound to different domain of IGF2BP3 (Supplementary Fig. 7j). In addition, only full-length IGF2BP3, rather the truncated mutants, enhanced *PDPN* mRNA stability (Supplementary Fig. 7k), arguing that circITGB6 modulated *PDPN* mRNA stability through its association with IGF2BP3. Considering that both circITGB6 and IGF2BP3 also regulates the expression of E-cadherin, N-cadherin and vimentin (Fig. 4g), we also measured the stability of these EMT-dependent transcripts upon wild-type IGF2BP3 or circITGB6/PDPN-interaction deficient and found IGF2BP3 had little effect on the stabilities (Supplementary Fig. 7l–o). Interestingly, Similar phenomena were also observed in cells with wide-type circITGB6 or its IGF2BP3-binding deficient variant (Supplementary Fig. 7p–s), thus excluding that circITGB6/IGF2BP3 facilitates EMT process by affecting the mRNA stabilities of EMT markers.

### circITGB6 induced EMT process and tumor metastasis through PDPN pathway

To explore if the functions of circITGB6 depend on PDPN pathway, we performed rescue assays by knocking down PDPN expression in circITGB6-overexpression cells. Immunoblot and Transwell migration assays showed that PDPN depletion repressed the change of circITGB6-mediated EMT process and cell migration (Supplementary Fig. 8a, b). Consistently, in vivo mouse experiments also demonstrated that PDPN depletion largely relieved the increased metastatic nodules in lungs formed by tail-vein injection after circITGB6-overexpression (Fig. 6g–i). Therefore, our results indicate that circITGB6 promotes tumor metastasis though enhancing *PDPN* mRNA stability.

In accordance to the aforementioned implication of PDPN in the circITGB6-facilitated EMT process, circITGB6 depletion repressed the increase of PDPN expression induced by TGFβ (Supplementary Fig. 8c). Considering that IGF2BP3 functions as a critical circITGB6-associating factor for the metastasis-related functions of circITGB6, we also performed a rescue assay by treated IGF2BP3-KD A549 cells with TGFβ. As expected, IGF2BP3 depletion dramatically blocked the upregulation of PDPN, cell migration ability, morphological transition and change of EMT markers upon TGFβ stimulation (Supplementary Fig. 8d–g). Similar phenomena were also observed in A549 cells with PDPN knockdown upon TGFβ stimulation (Supplementary Fig. 8h–j), agreeing with the previous findings that PDPN modulates TGFβ-induced EMT^47^. Collectively, these results highlighted the permissive effects of circITGB6/IGFBP3/PDPN pathway in TGFβ-triggered EMT process and cell migration.

Brain metastasis is quite commonly observed in solid tumors, with lung cancer (20–56% of patients) showing the highest tendency to conduct brain metastasize^48–50^. Therefore, to investigate the implication of circITGB6/PDPN pathway in brain metastasis, we performed three rounds of in vivo training to obtain highly brain-metastatic lung cancer cells (BrM) as previously described^51^ (Supplementary Fig. 8k). Transwell migration assays showed that BrMs exhibited the increased cell migration ability, compared to their parental cells (Supplementary Fig. 8l). Importantly, both circITGB6 and *PDPN* expressions were much higher in the BrMs than those in the parental cells (Supplementary Fig. 8m, n). Moreover, enhanced *PDPN* mRNA stability was also observed in BrM cells (Supplementary Fig. 8o), suggesting that circITGB6/PDPN pathway participates in brain metastasis of lung cancer. To further confirm the implication of circITGB6/PDPN pathway in tumor metastasis. We also explored the levels of TGFβ, circITGB6 and PDPN in the primary splenic tumors and metastatic liver lesions using by intra-splenic injections of SW620 cells. Compared to primary tumors, both circITGB6 and PDPN showed increased expression in metastatic tumors, accompanied with increased TGFβ abundance (Fig. 6j and Supplementary Fig. 8p). Interestingly, decreased E-cadherin and increased N-cadherin were also observed in metastatic tumors (Fig. 6j and Supplementary Fig. 8p). Collectively, these results suggested that the activated TGFβ/circITGB6/PDPN pathway in tumors metastasis.

### circITGB6 depletion effectively inhibits tumor metastasis

**Fig. 7:**
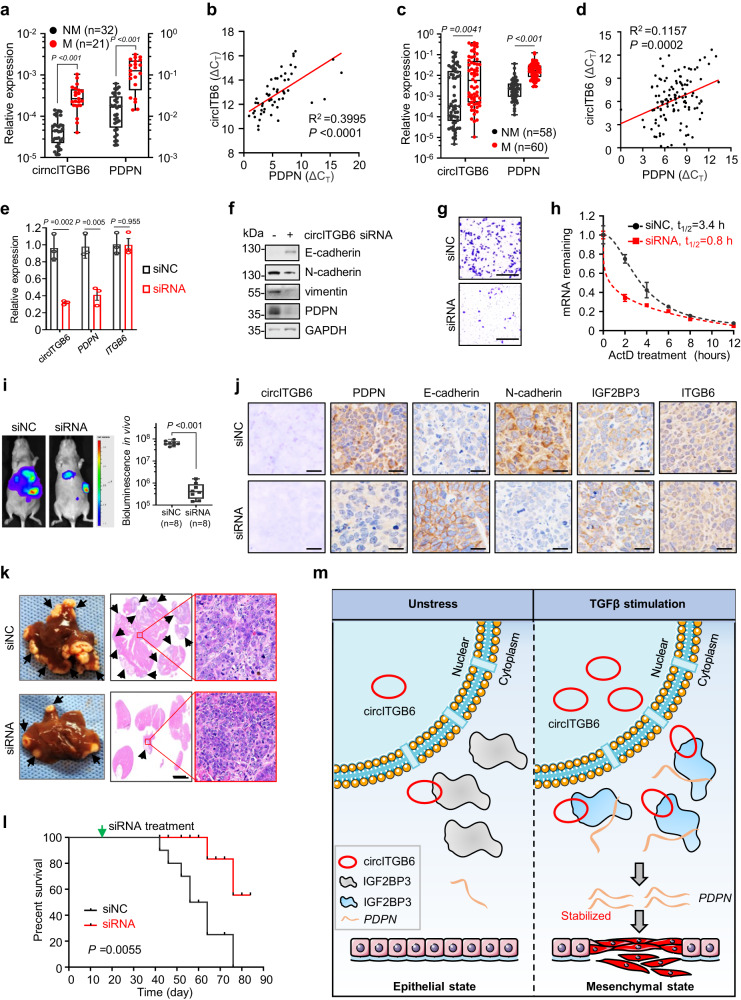
Targeting circITGB6 effectively inhibits tumor metastasis. **a**, **b** qPCR assays (**a**) and Pearson Correlation (**b**) of circITGB6 and *PDPN* expression in non-metastatic (NM) and metastatic (M) CRC tumor tissues (*n* = 53 samples, cohort 2). **c**, **d** qPCR assays (**c**) and Pearson Correlation (**d**) of circITGB6 and *PDPN* expression in non-metastatic and metastatic lung tumor tissues (*n* = 118 samples). The horizontal lines represent the median; the bottom and top of the boxes represent the 25th and 75th percentiles, respectively; and the vertical bars represent the range of the data (**a**, **c**). **e–h** SW620 cells were treated with PEI-coated control siRNA (siNC) or circITGB6 siRNA (siRNA). qPCR assays for measuring the expression of circITGB6, *ITGB6* and *PDPN* in SW620 cells (**e**), immunoblot assays of EMT markers (**f**), Transwell migration assays (**g**) and measurement of *PDPN* mRNA half-life (**h**) in SW620 cells. Scale bar: 20 μm. **i–I** Mice were intra-splenically injected with (2 × 10^6^) SW620 cells stably expressing luciferase to establish liver metastases. Two weeks later, mice were randomly divided into two groups and intravenously injected twice a week for additional 3 weeks with PEI-coated siNC or siRNA targeting circITGB6 (6 mg/kg body weight). Representative bioluminescence images of liver metastasis (**i**, *left*) and bioluminescence quantitation in vivo (**i**, *right*). The horizontal lines represent the median; the bottom and top of the boxes represent the 25th and 75th percentiles, respectively; and the vertical bars represent the range of the data. *n* = 8 mice per group. Representative images of the foci of circITGB6 (red point) and the IHC staining of PDPN, E-cadherin, N-cadherin, IGF2BP3 and ITGB6 in metastatic lesions (**j**). Photos of the livers and representative images of H&E staining metastatic liver tissues (**k**). the metastatic nodules were indicated by the black arrows (**k**). The metastatic nodules indicated by the black arrows. Scale bar: 20 μm (**j**); 4 mm (**k**). Kaplan–Meier curve for the overall survival of mice (**l**). Data represent mean ± sd (**e**, **h**) from three independent experiments, mean± s.e.m. (**i**). Significance of differences was determined by one-way ANOVA test (**a**, **c**, **i**), two-sided Pearson Correlation test (**b**, **d**), two-tailed Student’s *t* test (**e**) and the log-rank test (**l**). **m** Schematic illustration of circITGB6 molecular mechanism. Upon TGFβ stimulation, more circITGB6 are expressed to stabilize *PDPN* mRNA molecules through their interactions with IGF2BP3, thus facilitating EMT process and promoting tumor metastasis. Source data are provided as a Source Data file.

To further confirm the positive correction between circITGB6 and PDPN, we examined circITGB6 abundance by RNAscope assay and PDPN expression by Immunohistochemistry (IHC) assay in the primary tumor and metastatic liver lesions of mice intrasplenically injected with SW620 cells (mentioned in Fig. 5i). The result showed that overexpression of wide-type circITGB6 significantly increased PDPN expression, while the IGF2BP3 interaction-deficient circITGB6 mutant failed to upregulate PDPN expression (Supplementary Fig. 9a). Thus, circITGB6 interacts with IGF2BP3 to positively regulate PDPN expression in vivo. To explore the clinical relevance of circITGB6/PDPN pathway, a cohort of 53 CRC samples was employed to measure circITGB6 abundance and *PDPN* mRNA levels. The samples were divided into two groups according to the presence or absence of lymph node metastatic status. Compared to those without metastasis, both circITGB6 and *PDPN* mRNA showed higher expression in samples with lymph node metastasis (cohort 2, Fig. 7a). Notably, a significantly positive correction between circITGB6 and *PDPN* mRNA levels was observed among the CRC patients (cohort 2, Fig. 7b). We further confirmed the findings in a cohort of 118 lung cancer samples (Fig. 7c, d). Therefore, circITGB6 expression is positively correlated to *PDPN* abundance in a mouse liver metastatic model of CRC and human cancer biopsies.

Given the critical roles of circITGB6 in tumor metastasis (Fig. 2 and Supplementary Fig. 3 h, i) and its correlation with cancer progression (Fig. 1l–o and Fig.7a–d), we wonder if circITGB6 could be a potential therapeutic target for pro-metastatic tumor. To this end, siRNA specifically targeting the junction site of circITGB6 were generated and its selective attenuating effect on circITGB6 rather than *ITGB6* transcript was validated (Fig. 7e). Next, we evaluated the efficacy of PEI-coated circITGB6 siRNAs in highly metastatic SW620 cells. As expected, PEI-coated circITGB6 siRNA treatment effectively decreased *PDPN* expression at both mRNA and protein levels, and re-shaped the expression of EMT markers and also repressed cell migration (Fig. 7f, g). Notably, the half-life of *PDPN* mRNAs became shorter after circITGB6 depletion in SW620 cells (Fig. 7h), reinforcing our previous conclusion in Fig. 6. Next, we sought to evaluate the therapeutic potential of PEI-coated circITGB6 siRNAs for pro-metastatic tumors in vivo. For this goal, a mouse liver metastatic model was generated by intrasplenic injection with luciferase-labelled CRC SW620 cells. Two weeks later, mice were randomly divided into two groups and intravenously injected with PEI-coated siNC or circITGB6 siRNA twice a week for additional 3 weeks (Supplementary Fig. 9b). Intriguingly, compared to the control, circITGB6 siRNA treatment dramatically decreased the number and size of metastatic lesions in liver (Fig. 7i–k and Supplementary Fig. 9b), without causing obvious toxicity on main organs (Supplementary Fig. 9d). Most importantly, we observed a significant improvement of survival in mice treated with PEI-coated circITGB6 siRNA compared to the PEI-coated siNC group (Fig. 7l). Taken together, our results demonstrate that targeting circITGB6 via siRNA is a promising strategy for cancer therapy.

## Discussion

Although the role of circRNAs in tumor metastasis was recently recognized^14–16^, the detailed implication of circRNAs in TGFβ-mediated EMT process is still obscure. In this study, we identified a conserved circRNA called circITGB6 that was robustly induced by TGFβ (Fig. 1a–d and Supplementary Fig. 2i), promoted EMT process and tumor metastasis in CRC and lung cancer (Figs. 2 and 3). Mechanistically, circITGB6 promotes tumor metastasis through its direct interaction with IGF2BP3 (Figs. 4 and 5), leading to activation of IGF2BP3 and consequent stabilization of its downstream transcript, *PDPN* mRNA (Figs. 6 and 7m). Moreover, the activated circITGB6/IGF2BP3/*PDPN* axis is closely correlated to metastasis status in lung cancer patients (Fig. 7c, d), increased lymph node metastasis (Fig. 7a, b) as well as worse prognosis in CRC patients (Fig. 1m), reinforcing the critical role of circITGB6 in tumor metastasis. Intriguingly, PEI-coated circITGB6 siRNAs demonstrated a satisfactory benefit for mice bearing metastasis (Fig. 7i–l). Collectively, our findings support the important role of circITGB6 in TGFβ signaling and also highlight the promise of targeting circITGB6 for cancer therapy.

As a critical contributor to EMT reprogramming, TGFβ pathway equips cancer cells with the migration potential to disseminate from their original lesions to establish metastasis^52^. To achieve this goal, many effectors including EMT-transcriptional factors^53^, miR-200 family^54–56^ and lncRNA-ATB^28^ were recently identified to participate in EMT process and tumor metastasis, highlighting complexity of TGFβ pathway. Although dysregulated circRNAs were identified in metastatic tumors, the involvement of circRNAs in TGFβ-mediated EMT process remains obscure. In this study, we identified circITGB6 as a pivotal factor of TGFβ network, which elicits EMT process and endow cancer cells with metastatic properties. Upon TGFβ stimulation, circITGB6 was significantly upregulated at a time- and dose-dependent manner (Fig. 1c, d). In various in vitro and in vivo models, enforced circITGB6 expression dramatically promotes cell migration and tumor metastasis (Figs. 2 and 3), supporting that circITGB6 functions as a metastasis-specific factor. Besides, circITGB6 knockdown robustly delayed the EMT phenotype triggered by TGFβ (Fig. 2f–i). Interestingly, circITGB6 seemed not affect the expressions of canonical EMT-related regulators (Supplementary Fig. 4a, b) or the TGFβ downstream effectors (Supplementary Fig. 4h). Notably, circITGB6 has minimal effect on the expression of ITGB6 (Supplementary Fig. 3d–f), which activates the latent TGFβ and endow breast cancer immunotherapy resistance^57^. Still, circITGB6 overexpression could also promote cell migration upon ITGB6 depletion (Supplementary Fig. 4e–g), suggesting that circITGB6 promotes EMT independent of ITGB6 pathway. In regard to the modulatory mechanism on TGFβ-induced circITGB6 increase, the simultaneously upregulation of circITGB6 and its host transcript ITGB6 suggests a possible implication of transcriptional activation (Supplementary Fig. 1k). Further efforts are required to identify the specific transcription factors that are responsible for TGFβ-induced circITGB6 activation.

Accumulating studies have recently reported that circRNAs exert their biological functions through interacting with RNA-binding proteins (RBPs) to regulate gene expression^28,58–60^. Here, we identified IGF2BP3 as a TGFβ-responsive circITGB6 interactor (Fig. 4a–f), whose depletion abolished the metastasis-promoting effect of circITGB6 (Fig. 4 g, h), consistent with the positive correlation between IGF2BP3 and tumor metastasis^43,61^. In detail, the ‘CAUU’ motif within circITGB6 is indispensable for its direct interaction with IGF2BP3, as mutations of this motif from ‘CAUU’ to “GUUU” destroyed circITGB6/IGF2BP3 interaction (Fig. 5a–g). Consequently, the circITGB6/IGF2BP3 interaction increases the stability of *PDPN* mRNA, which encodes a key metastasis-promoting factor^6,7^. PDPN favors metastasis via binding ERM proteins to remodel actin cytoskeletons^44,62^. PDPN depletion dramatically repressed TGFβ-induced EMT process^47^ (Supplementary Fig. 8h–j), pronouncing that the implication of PDPN for EMT process and tumor metastasis. Facilitated EMT process and migration as a consequence of upregulated PDPN abundance in the selected brain-metastasis cell model (Supplementary Fig. 8k–o) and liver metastatic mouse model in vivo (Fig. 6j) further confirmed that PDPN promoted tumor invasion^63^. Notably, different IGF2BP3 KH domain was identified to interact with circITGB6 and *PDPN* mRNA, respectively, which rule out the possibility that circITGB6 competes with *PDPN* mRNA for IGF2BP3 interaction (Fig. 6e and Supplementary Fig. 7j, k). Consistently, our data showed that *MMP9*, another well-known target of IGF2BP3^64,65^, was also induced by circITGB6 (Fig. 6a), implying that circITGB6 association may change the protein confirmation of IGF2BP3 to promote its interaction with downstream mRNA cargos. However, such sophisticated molecular mechanism needs to be further explored.

Metastasis remains the most important cause of cancer-related death. Still, in current anticancer therapies, a potent and clinically practicable category of anti-invasion and anti-metastatic drugs is under urgent demand^66,67^. Through complementary base pairings, small interfering RNA is capable of silencing specific gene expression of both mRNAs and ncRNAs^68,69^. The expansion of tractable target spectrum by siRNAs is one of the most attractive advantages to targeting ncRNAs which can be hardly target by the traditional small chemical molecules and antibody drugs. The recent approval of patisiran^70^ and givosiran^71^ by FDA further pronounce the feasibility of RNAi strategy. Based on the critical role of circITGB6 in promoting tumor metastasis (Figs. 2, 3) as well as the specific high expression of circITGB6 in metastatic cancer samples (Figs. 1n, 6j, 7a, c and Supplementary Fig. 2c), a PEI-coated siRNA compounds specifically decreasing circITGB6 expression (Fig. 7e) were generated to explore its therapeutic potential for metastatic cancer in widely used mouse models of CRC with liver metastasis. To our surprise, the PEI-coated circITGB6 siRNA demonstrated satisfactory benefit for mice bearing metastasis, as evidenced of the decreased the number and size of metastatic lesions in liver (Fig. 7i, k) and prolonged the survival mice bearing metastasis (Fig. 7l). Moreover, such treatment was well-tolerated as no obvious toxicity was observed in mice (Supplementary Fig. 9d). Notably, there are candidate PAM sequences around the back-splice site of circITGB6, which make CRISPR/Cas-based approach effective pharmacological targeting of circITGB6 expression. In summary, our study underscores the feasibility and therapeutic potential of manipulating circITGB6 expression to treat metastatic cancers.

## Methods

### Patients and ethical statement

All procedures involving the use and care of animals were conducted in compliance with standard procedures approved by the Institutional Animal Care and Use Committee of West China Hospital, Sichuan University. The procedures to obtain all human material were approved by the Ethics Committee of West China Hospital, Sichuan University (2018(280); 2019(338)). The CRC and lung tumor tissues used in this study were obtained from West China Hospital, Chengdu, with informed written consent from patients. There was no bias in the selection of patients. The cDNA microarray of human colon cancer tissues (cohort 1, Cat#cDNAHColA095Su02) was purchased from Shanghai Outdo Biotech Co., Ltd, and the clinical information of these patients is available in Supplementary Table 2.

### Animal experiments

All mouse procedures were approved by the Institutional Animal Care and Use of West China Hospital, Sichuan University. The 4–6-week-old female nude mice were purchased from Beijing HFK Bioscience. Animals were housed under the following conditions: temperatures of 21.0–23.0 °C, 40–60% humidity, 10–15 fresh air exchanges hourly, and a 12:12 h light: dark cycle (lights were on from 06:00–18:00). For the liver metastasis model, 1 × 10^6^ SW620 cells (2 × 10^6^ SW620 cells for siRNA in vivo delivery experiments) suspended in 100 μL PBS were injected into beneath the inferior hemispleen of nude mice. For the lung metastasis model, 1 × 10^6^ cells were injected into the tail-vein of nude mice. Six weeks after cell injection, the mice were sacrificed for experiments. For systemic spread of tumor by intracardiac injection. 1 × 10^5^ cells suspended in 100 μL PBS were injected into left cardiac ventricle of nude mice. Bioluminescence images of tumor-bearing mice were acquired with IVIS Spectrum (PerkinElmer) at 15 min after intraperitoneal injection of D-luciferin (15 mg/kg, Promega) and analyzed using the LivingImage 3.2 software package (Caliper LifeSciences). The maximal tumor size permitted by the IACUC of Sichuan University is 20 mm at the largest diameter in mice. No weight loss was observed in mice with lung metastasis, <5% weight loss was permitted for mice with liver metastasis or systemic metastasis. Mice were euthanized by rapid cervical dislocation at the end of the experiment (8 weeks after intrasplenic or intracardiac injection, 6 weeks after tail-vein injection), after which tumors and major organs were collected by using surgical scissors. The lungs and livers were removed and fixed with 4% formalin. The numbers of metastatic nodules in the lungs and livers were carefully examined.

### Cell culture and transfection

Py2T cells were isolated from a breast tumor of a FVB/NJGpt-Tg (MMTV-PyMT)/Gpt female mouse (GemPharmatech, Strain NO.T004993). Murine breast cancer cell Py2T, Human embryonic kidney cell HEK293T, Breast cancer cell MCF7, CRC cell SW620, SW480, HCT116, HT29 and pancreatic cancer cell Capan-2 were cultured in DMEM supplemented with 10% FBS (Gibco) and 100 mg/mL streptomycin (Gibco). human lung cancer cells A549 and HCC827 were cultured in RPMI 1640 plus 10% FBS and 100 mg/mL streptomycin. Human mammary epithelial cell MCF-10A was maintained in DMEM/Ham’s F-12 nutrient mixture (Gibco) with 5% horse serum (Gibco), 20 ng/ml EGF (Peprotech), 10 μg/ml insulin, 0.5 μg/ml hydrocortisone (Sigma) and 100 mg/mL streptomycin. A549 and MCF-10A were treated with 5 ng/ml TGFβ (Peprotech) for 72 h. Transfection was performed with Lipofectime 2000 (Thermo Scientific) according to the manufacturers’ instructions. In vivo delivery of siRNAs was performed with in vivo-jetPEI (Polyplus) according to the manufacturers’ instructions.

### High-throughput RNA-sequencing for circRNA

To identify the differentially expressed circRNA in MCF10A cell with or without 5 ng/ml TGFβ treatment for 72 h, total RNAs were extracted with TRIZOL reagent (Invitrogen). circRNA-sequencing libraries were generated using rRNA Depletion Kit and VAHTS Universal V6 RNA-seq Library Prep Kit (Vazyme #NRM604-01) according to the manufacture’s instruction. circRNAs were identified by CIRC2 algorithm and annotated through blasting the sequence in circBase. We chose circRNAs with a fold change ≥1.5 and a *P* value < 0.05 in a comparison between untreated and TGFβ-treated cells as significantly differentially expressed circRNAs.

### Plasmid construction

pLaccase2 plasmid was obtained from Dr. Jeremy E. Wilusz as circRNA overexpression backbone. circITGB6 overexpression plasmid was produced by inserting circITGB6 sequence amplified from MCF10A cDNA (with TGFβ treatment) into pLaccase2 plasmid with One-Step Cloning Kit (Vazyme). For circITGB6-CD plasmid, the splicing acceptor sequence was destroyed with QuikChange Site-Directed Mutagenesis Kit (Agilent Technologies). circITGB6-mut plasmid was cloned by replacing the IGF2BP3 interaction-deficient circITGB6 mutant sequences in the pLaccase2-circITGB6 plasmid. IGF2BP3 cDNA (NM_006547) was purchased from Miaolingbio. Core EMT-related TFs, full-length and truncated IGF2BP3 plasmids with Flag-tag were generated by inserting indicated coding-regions into the vector pCDH-CMV-MCS-EF1-puro (System Bioscience). The primers were shown in Supplementary Table 5.

### qPCR and RT-PCR

Total RNAs were extracted using Trizol Reagent (Takara). 1 μg of total RNAs were subjected to cDNA synthesis with PrimeScript^TM^ RT reagent Kit (Takara) according to the manufacturers’ instruction. qPCR was performed using SYBR Green 2xTaq mix (Takara) and analyzed with QuantStudio 6 Operating Software (Life technologies).

### Determination of mRNA half-life

Cells were treated with 5 µM actinomycin D (ActD; Sigma) for the indicated times, followed by RNA extraction and cDNAs synthesis. Remaining RNA levels at indicated time points were normalized to the level at the beginning (0 h). One-phase exponential decay curve analysis (GraphPad Prism) was used to assess mRNA decay kinetics.

### RNase R treatment

5 μg of total RNAs were incubated with 10 unit of RNase R (Epicentre Technologies) at 37 °C for 15 min, and then subjected to RT-PCR or Northern blotting to examine RNA levels of circITGB6 and other indicated mRNAs.

### Cytosolic/Nuclear fractionation

1×10^7^ cells were re-suspended in the hypotonic buffer (25 mM Tris-HCl, PH 7.4, 1 mM MgCl_2_, 5 mM KCl, 0.5% NP-40) and incubated on ice for 5 min. After centrifuging the cells at 5000 *g* for 5 min, the supernatant was collected as the cytosolic fraction. The pellets were washed twice with hypotonic buffer, and then re-suspended in nuclear buffer (20 mM HEPES, pH 7.9, 400 mM NaCl, 1 mM EDTA, 1 mM EGTA, 1 mM DTT, 1 mM PMSF). After incubation on ice for 20 min, the sample was centrifuged for collecting the nuclear fraction. GAPDH, *Actin*, Lamin A/C and *U6* served as the cytosolic or nuclear fraction controls.

### RNA FISH

FISH assays were performed to detect RNA levels using Ribo^TM^ Fluorescent in Situ Hybridization Kit (RiboBio) according to the manufacturer’s protocol. The Cy3-labeled probes complementary to *U6*, *18* *S* rRNA, or the back-splice site of circITGB6 (Supplementary Table 5) were used for hybridization, and the signal was detected with a confocal laser scanning microscope (Zeiss LSM510).

### RNAscope in situ hybridization

RNAscope in situ hybridization was performed to detect single RNA molecules of circITGB6 using The BaseScope™ Reagent Kit v2-RED (Advanced Cell Diagnostics) according to the manufacturer’s instructions. The circITGB6 probe (1227451-C1) was designed against nucleotides in the back-splice site of circITGB6.

### Immunofluorescence assays

A549 Cells grown on the confocal dish (Corning) were fixed with 4% paraformaldehyde for 20 min at room temperature, then washed gently three times with ice-cold PBS and permeabilized with 0.2% Triton X-100 in PBS for 20 min at room temperature. Following 1 h blocking in 1% BSA (dissolved in TBS-Triton X-100 solution) at room temperature, coverslips were incubated overnight at 4 °C with anti-IGF2BP3 antibodies (Abcam, Cat#ab177942, 1:100 dilution) diluted in blocking buffer containing 0.2% Triton X-100. Following three 5 min washes in PBS, coverslips were incubated with secondary antibodies (anti-rabbit IgG, Bioss, #bs-0295G-AF488, 1:300) simultaneously diluted in blocking buffer containing 0.2% Triton X-100 for 1 h at room temperature in the dark, then incubated with DAPI for 15 min at room temperature in the dark. Following three 5 min washes with PBS, coverslips were mounted in Immu-Mount (Thermo Scientific) and imaged on a Zeiss LSM510 meta upright confocal microscope.

### Immunohistochemistry

Tissue samples were embedded in paraffin and cut into serial 4 µm-thick sections. The slides were incubated at 65 °C until the paraffin melts, followed by deparaffinization in three changes of xylene and rehydrated through 5 min incubations in 100, 95, 85, and 75% ethanol solutions. For histological examination, sections were stained with hematoxylin and eosin for 3–5 min. For Immunohistochemical staining, antigens were retrieved in a 20 mM sodium citrate buffer boiled for 15 min. The tissue sections were then cooled in ice bath for at least 20 min and rinsing in PBS three times. Endogenous peroxidase activity was blocked using 1% H_2_O_2_ for 15 min, followed by blocking of the nonspecific binding sites with 5% goat serum for 30 min. Subsequently, blocked sections were incubated with indicated antibody (anti-PDPN, 1: 400 dilution; anti-TGFB1, 1:400 dilution; anti-IGF2BP3, 1:100 dilution; anti-E-cadherin, 1:400 dilution; anti-N-cadherin, 1:125 dilution and anti-ITGB6, 1:200 dilution) overnight at 4 °C in a wet box. Next day, rinse the tissue section three times in PBS for 5 min each time and incubated with HRP-conjugated second antibody at room temperature for 45 min. The tissue sections were stained with diaminobenzidine tetrahydrochloride (DAB) for 30 s and wash with distilled water three times for 5 min each time. Finally, the sections were dehydrated and sealed with neutral gum. The results were analyzed using the Inform software (Akoya Biosciences). Three high-power fields (×20) of each sample were selected at random.

### Northern blotting

Total RNAs were subjected to denaturing 4% acrylamide gel electrophoresis and subsequently transferred onto Hybond-N^+^ membrane (GE Healthcare) with 0.5 × TBE buffer. Then the membrane was UV cross-linked and prehybridized with the ULTRAhyb™ Ultrasensitive Hybridization Buffer (Invitrogen, cat#AM8669) for 30 min, followed by incubation with biotin-labeled DNA probes at 42 °C overnight. β-actin mRNA or 18 S rRNA was used as the loading control. Signals were detected with HRP-conjugated streptavidin. DNA probes were listed in Supplementary Table 5.

### Cell migration assays

Cells were suspended in serum-free medium and seeded into the upper chamber, while complete medium was added into the bottom chambers as chemoattractant. After incubation at 37 °C for 24 h, the migrated cells were fixed with 4% paraformaldehyde for 20 min and stained with 1% Crystal violet (Sigma) for another 20 min.

### Cell proliferation assays

1000 cells were seeded in each well of 96-well plates and culture for indicated times. Then 10 μL MTT solution was added into each well, followed by incubation at 37 °C for 3 h and dissolve with 150 μL DMSO solvent for measuring absorbance at 590 nm. For colony formation assay, 3000 cells ware seeded in each well of 6-well plates and cultured for 10 days. The colonies were fixed and stained.

### Western blot

Proteins were extracted with RIPA lysis buffer (Beyotime Biotechnology, cat# P0013B) plus protease and phosphatase inhibitor cocktail (Roche, cat# PPC1010), and quantified by Pierce™ BCA Protein Assay Kit (Thermo Scientific, cat# 23225). Equal amounts of proteins were separated by SDS–PAGE and transferred onto PVDF membrane (GE health). After incubation with the primary antibody at 4 °C overnight, the membrane was washed with TBS-T buffer three times and incubated with HRP-conjugated anti–mouse or anti-rabbit IgG for 1 h at room temperature. Signal on the membrane was detected with ChemiDoc Imaging Systems (Bio-Rad). Antibodies used in this study: anti-IGF2BP3 (#ab177942, 1:2000), anti-TGFβR1(#ab235578, 1:1000) from Abcam; anti-LaminA/C (Abways, Cat#A1952, 1:2000); anti-SMAD7 (ABclonal, Cat#A12343, 1:1000); anti-flag (Abmart, Cat#M20008, 1:5000); anti-SRSF1 (HuaBio, Cat#ET7107-70, 1:1000); anti-PDPN (#11629-1-AP, 1:1000), anti-YBX1 (#20339-1-AP, 1:1000), anti-QKI(#13169-1-AP, 1:1000), anti-ITGB6 (#19695-1-AP, 1:1000), anti-TGFB1 (#21898-1-AP, 1:1000), anti-SERPINE1 (#13801-1-AP, 1:1000), anti-ZEB1 (#21544-1-AP, 1:2000) from Proteintech; anti-SMAD2/3 (#8685, 1:1000), anti-phospho-SMAD2/3 (#8828, 1:1000), anti-slug (#9585, 1:1000), anti-Snail1 (#3879, 1:1000), anti-Twist1 (#69366, 1:1000), anti-Vinculin (#4650, 1:1000), anti-E-cadherin (#3195, 1:1000), anti-GAPDH (#8884, 1:5000), anti-N-cadherin (#13116, 1:1000), and anti-Vimentin (#5741, 1:1000) from Cell Signaling.

### In vitro cyclization of circRNA

Linear RNAs were transcribed using MAXIscript™ T7/T3 Transcription Kit (Thermo Scientific) and Biotin RNA Labeling Mix (Roche) according to the manufacturer’s instructions. Linear RNAs were incubated with the DNA splints (molar ratio = 1:1.5) at 95 °C for 5 min in the annealing buffer (100 mM NaCl, 10 mM Tris-HCl pH 7.5), and then cooled down to room temperature and ligated with T4 DNA ligase (NEB) at 16 °C overnight to form circRNAs. After treatment with RNase R (Epicentre) and DNase I (Thermo Scientific) at 37 °C for 20 min to remove DNA templates and linear transcripts, circRNAs were purified by phenol-chloroform extraction. The primers and DNA splints are shown in Supplementary Table 5.

### RNA pull-down assay

#### Pull-down assay with DNA probes

A549 cells were transfected with 100 pmol DNA probes complementary to the back-splice site of circITGB6 or *PDPN* 3′UTR region. 24 h later, cells were washed with PBS and lysed with the lysis buffer (150 mM NaCl, 20 mM Tris-HCl, pH 7.5, 1.5 mM MgCl_2_, 0.5% NP-40, 0.5% Triton X-100, 10% glycerol, RNase inhibitors (Thermo Scientific), protease and phosphatase inhibitor cocktails). Pre-treated streptavidin magnetic beads were incubated with cell lysates at 4 °C for 1 h, followed by washing five times with the washing buffer (20 mM Tri-HCl, pH7.5, 1 mM EDTA and 450 mM NaCl) and elution with Laemmli sample buffer. The circITGB6- or *PDPN* mRNA- interacting proteins were subjected to SDS-PAGE and visualized by coomassie blue staining or immunoblot analysis. Protein bands were excised and identified by LC-MS/MS mass spectrometry and Proteome Discoverer software (version 1.4; Thermo Scientific).

#### Pull-down assay with in vitro cyclized circRNA

Equal amounts (2 μg) of sense and antisense circRNAs were denatured and renatured to form an appropriate secondary structure. Cell lysates were incubated with renatured circRNAs at 4 °C for 4 h, followed by mixing with pre-treated streptavidin magnetic beads. After washing five times with the wash buffer, the bound proteins were subjected to immunoblot analysis.

#### Pull-down assay with RNA probes

200 pmol of biotin-labeled RNA probes were incubated with streptavidin magnetic beads at 4 °C for 1 h. After washing once with the washing buffer (20 mM Tri-HCl, pH7.5, 1 mM EDTA and 450 mM NaCl), the resultant beads were incubated with cell lysates at 4 °C for 3 h and washed five times. Then the bound proteins were subjected to immunoblot analysis.

#### RNA-IP assay

Cells were lysed in pull-down lysis buffer containing protease inhibitors and RNase inhibitors on ice for 15 min, followed by homogenization and centrifugation, the supernatant was incubated with anti-IGF2BP3 and equivalent rabbit IgG for 4 h with gentle rotation. The mixtures were precipitated by Protein A/G Agarose beads (Millipore, #IP10) at 4 °C for additional 1 h. The beads were next washed three times. The beads-bound proteins were further analyzed using western blotting. The immunoprecipitated RNA was purified by Trizol Reagent and applied to qRT-PCR analysis.

#### Protein purification

IGF2BP3 coding-region was amplified by RT-PCR and cloned into pGEX-6P-1-GST plasmid at *Bam*HI and *Not*I sites to obtain the recombinant plasmid pGEX-GST-IGF2BP3, which was transformed into *E.coli* BL21 (DE3). IGF2BP3 expression was induced with 0.1 mM IPTG at 16 °C for 24 h and subsequently purified with GST resins. After extensive washing, IGF2BP3 was eluted with the elution buffer (20 mM Tris-HCl pH 8.0, 150 mM NaCl and 1 mM DTT).

#### RNA electromobility shift assays

Wild-type (WT) or mutant (mut) RNA probes (Supplementary Table 5) were synthesized by Sangon Biotech. The recombinant IGF2BP3 proteins were incubated with the probes in binding buffer (10 mM HEPES (7.3), 20 mM KCl, 1 mM MgCl_2_, 1 mM DTT). The mobility shift assay was performed using a Light Shift Chemiluminescent RNA EMSA Kit (Beyotime) according to the manufacturer’s protocol.

#### Competitive binding assays

The mixture of recombinant IGF2BP3 proteins (1 μM) and biotin-labeled circITGB6 (1 μM) were incubated with non-biotin control NC or gradient-concentration M1, M2 or M1-mut RNA probes in binding buffer (150 mM NaCl, 20 mM Tris-HCl, pH 7.5, 1.5 mM MgCl_2_, 1 mM DTT, 10% glycerol) at 4 °C for 4 h with gentle rotation. Pre-treated streptavidin magnetic beads were added and incubated for additional 1 h. The beads were next washed three times. The beads-bound proteins were further analyzed using western blotting. Protein levels were quantified with Image J software and normalized to control NC samples.

### Statistics and reproducibility

Statistical analyses were carried out using SPSS 26.0 statistical software package (IBM) or GraphPad Prism (version 9.0). For imaging experiments, the field of view was randomly selected for statistical analysis and the representative image was shown. The blotting experiments performed once, however the critical results or conclusions were repeated by adding additional experimental arms (CD or mut group) in the other parts of the article. All qPCR experiments were repeated independently with similar results for three times, and data are presented as mean ± standard deviation (sd) or standard error of the mean (s.e.m). The statistical significance of differences was evaluated by two-tailed Student’s *t* test or one-way ANOVA test. Overall survival was assessed with the Kaplan−Meier analysis. A log-rank test was used to analyze the differences between groups. The correlations between circITGB6 and *PDPN* expression in CRC patients were calculated by *Pearson* correlation analysis. *P* values < 0.05 were considered as statistical significance.

### Reporting summary

Further information on research design is available in the Nature Portfolio Reporting Summary linked to this article.

### Supplementary information

Supplementary information file

Reporting Summary

### Source data

Source Data

## Data Availability

The raw RNA-seq data generated in this study have been deposited in Gene Expression Omnibus (GEO) under the accession number GSE165576. The proteomics data generated in this study have been deposited in the ProteomeXchange Consortium (http://proteomecentral.proteomexchange.org) via PRIDE partner repository with the dataset identifier PXD043341. The remaining data are available within the paper, Supplementary Information or Source Data file. Source data are provided with this paper.
