# Supplementary material for: A circular RNA activated by TGFβ promotes tumor metastasis through enhancing IGF2BP3-mediated PDPN mRNA stability
Source: Nat Commun. 2023 Oct 28;14:6876. doi: 10.1038/s41467-023-42571-1 (PMC10613289; doi:10.1038/s41467-023-42571-1)
Supplement: Supplementary file 1 — Supplementary information file [file 41467_2023_42571_MOESM1_ESM.pdf]

Supplementary Information:

A Circular RNA Activated by TGFβ Promotes Tumor Metastasis through Enhancing IGF2BP3-mediated *PDPN* mRNA Stability

Ke Li[1,3], Jiawei Guo[1,3], Yue Ming[1], Shuang Chen[1], Tingting Zhang[1], Hulin Ma[1], Xin Fu[1], Jin Wang[1], Wenrong Liu[1], Yong Peng[1,2,*]

Supplementary Figures:

Supplementary Fig.1

Supplementary Fig.2

Supplementary Fig.3

Supplementary Fig.4

Supplementary Fig.5

Supplementary Fig.6

Supplementary Fig.7

Supplementary Fig.8

Supplementary Fig.9

Supplementary Tables:

Supplementary Table 1

Supplementary Table 2

Supplementary Table 3

Supplementary Table 4

Supplementary Table 5

Supplementary Figures:

Supplementary Fig.1

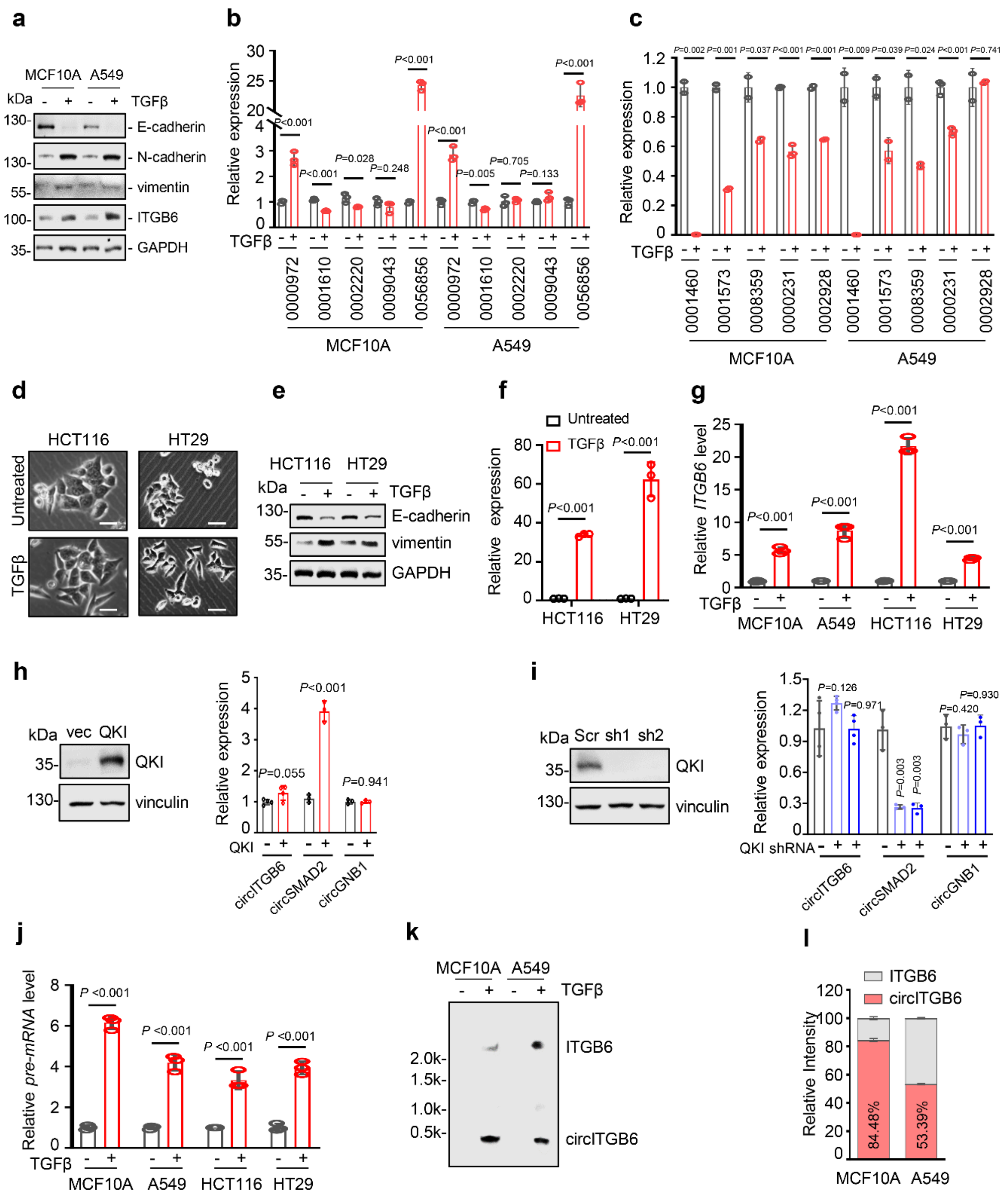

Supplementary Fig. 1 | circITGB6 is obviously induced by TGFβ. a Immunoblotting assays for the protein levels of EMT-related makers and ITGB6 in MCF10A and A549 cells with or without 5 ng/mL TGFβ treatment for 72 hours. GAPDH was used as an internal control. **b,c** qPCR assays for screening the TGFβ-response upregulated **(b)** and downregulated **(c)** candidate circRNAs,. **d-f** Representative phase contrast images **(d)**, EMT markers expression **(e)** and circITGB6 expression **(f)** in CRC HCT116 and HT 29 cells with or without 5 ng/mL TGFβ treatment for 72 hours. Scale bar: 10 μm **(d)**. **g** qPCR assay for the expression of *ITGB6* mRNA in MCF10A, A549 cells, HCT116 and HT29 cells with or without TGFβ treatment.

h,i qPCR assays for examining the effects of QKI overexpression **(h)** or knockdown **(i)** on circITGB6 levels in A549 cells. circSMAD2 was used as the positive control. circGNB1 was used as the negative control. **j** qPCR assay for the expression of *ITGB6* pre-mRNA (**j**) in MCF10A, A549 cells, HCT116 and HT29 cells with or without TGFβ treatment. **k** Northern blotting of circITGB6 and ITGB6 in MCF10A and A549 cells with TGFβ treatment. **l** Percentage of circITGB6 and ITGB6 levels in A549 and MCF10A cells with TGFβ treatment. Relative intensity derived from signal quantification of three independent experiments (mean ± sd). Data represent mean ± sd from three independent experiments (**b,c,f-j**). Significance of differences was determined by unpaired, two-tailed Student's *t*-test (**b,c,f-j**). Source data are provided as a Source Data file.

Supplementary Fig.2

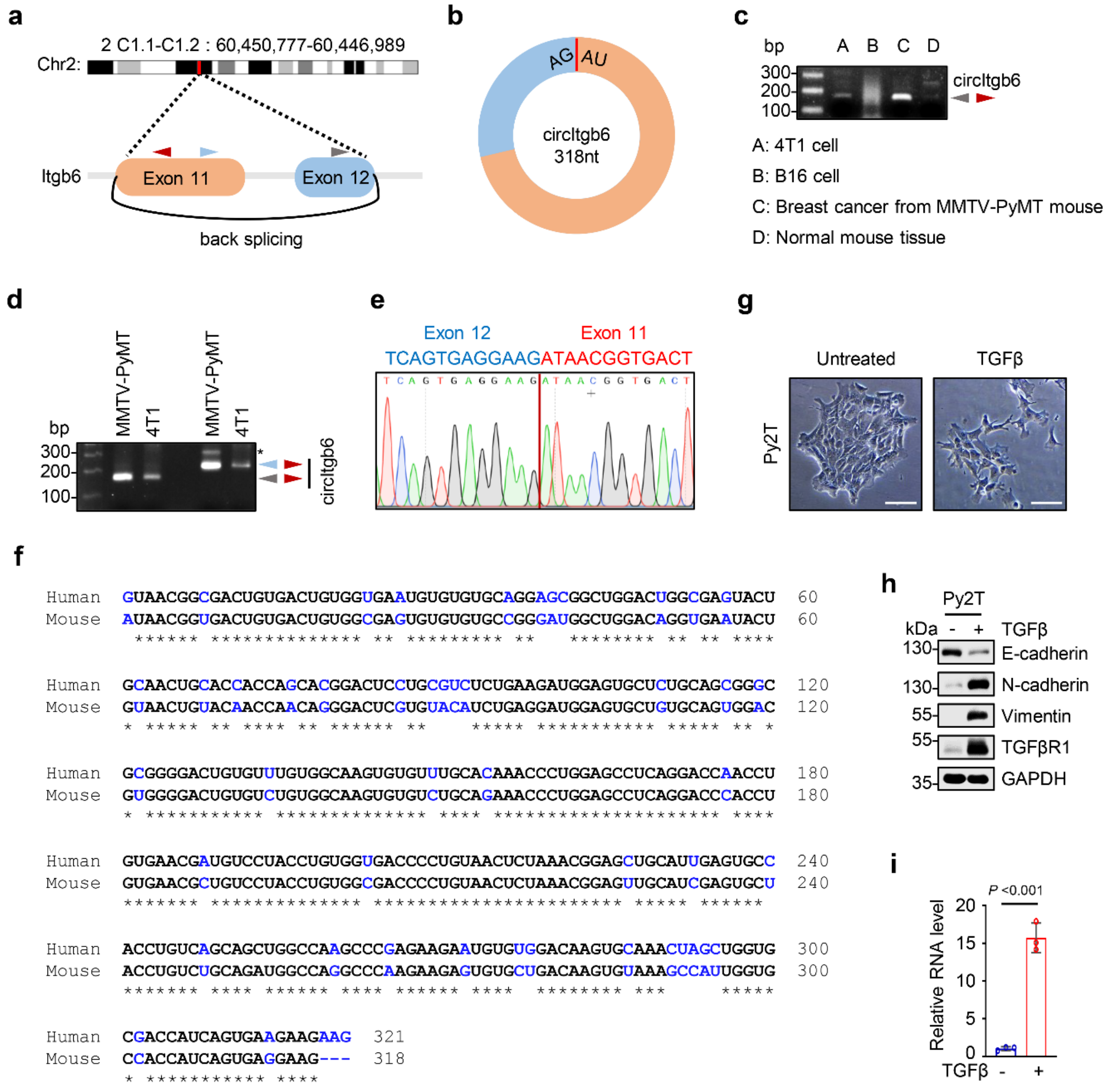

Supplementary Fig. 2 | circITGB6 is highly conserved cross human and mouse. a,**b** The genomic loci of mouse circItgb6 **(a)** and the predicted back-splice exons for circItgb6 **(b)**. **c**,**d** circItgb6 were amplified from mouse cell lines or mouse tissues by RT-PCR assays using independent divergent primers (indicated by colored triangle in (**a**), respectively). **e** Validation of head-to-tail splicing (indicated by red line) between Itgb6 exon 11 and exon 12 by Sanger sequencing. **f** The aligned sequences of human circITGB6 and mouse circItgb6 transcripts, and the different nucleotides were shaded as bule. **g-i** Representative phase contrast images **(g)**, EMT markers expression **(h)** and circItgb6 expression **(i)** in Py2T cells with or without 5 ng/mL TGFβ treatment for 72 hours. Scale bar: 20 μm. Data represent mean ± sd from three independent experiments **(i)**. Significance of differences was determined by unpaired, two-tailed Student's *t*-test (**i**). Source data are provided as a Source Data file.

Supplementary Fig.3

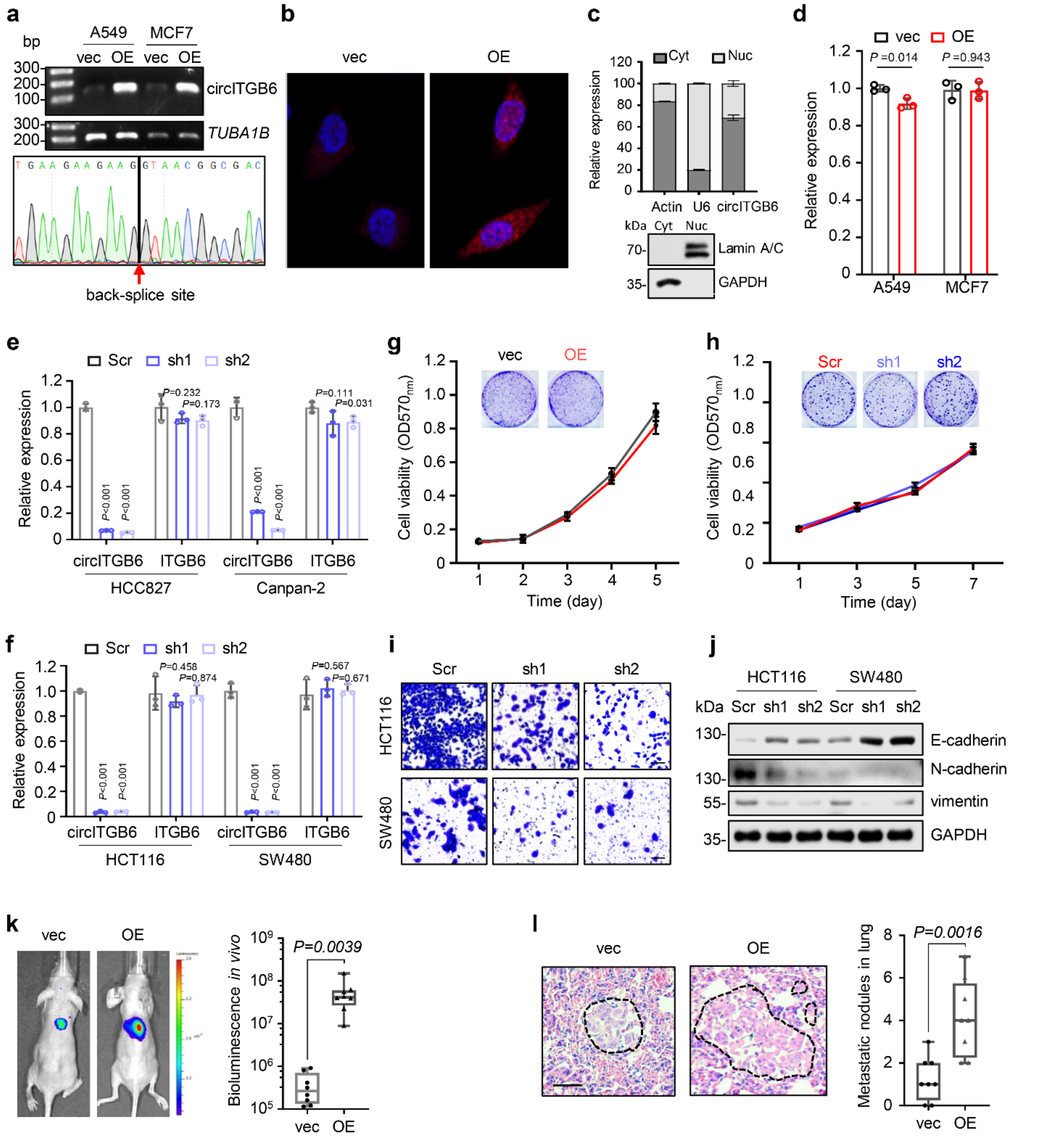

Supplementary Fig. 3 | circITGB6 has no effect on cell growth. a Sanger sequencing and RT-PCR assays to show successful overexpression and correct circularization (indicated by the red arrow) of circITGB6 overexpression in A549 cells. Vec, vector; OE, overexpression. **b,c** Subcellular distributions of circITGB6 identified by FISH assays (**b**) and cytoplasmic/nucleus fractionation assays (**c**) in A549 cells with or without circITGB6 overexpression. **d** qPCR assays showing circITGB6 overexpression has little effect on expression of its host gene ITGB6**. e,f** qPCR assays for measuring the expression of circITGB6 and ITGB6 in various cancer cells with circITGB6 knockdown. **g,h** MTT assays and colony formation assays for cell proliferation in A549 cells with circITGB6 overexpression (**g**) HCC827 cells with circITGB6-knockdown (**h**). **i,j** Transwell migration assay (**i**) and immunoblotting assay for the expression of EMT markers (**j**) in HCT116

and SW480 cells with or without circITGB6 knockdown. **k,l** A549 cells (1 × 10^6) with stable circITGB6 overexpression were tail-vein injected for metastasis assays (n = 8 mice per group). **k** Representative bioluminescence imaging of lung metastasis and quantitation of bioluminescence were presented. **l** Representative images for H&E staining of mouse lung tissues (left) and the numbers of observed metastatic nodules in the lung surface (right). Scale bar,20 μm. The horizontal lines represent the median; the bottom and top of the boxes represent the 25th and 75th percentiles, respectively; and the vertical bars represent the range of the data. Data represent mean ± sd from three independent experiments (**d-h**); Data represent mean ± e=s.e.m (**k,l**). Significance of differences was determined by unpaired, two-tailed Student's *t*-test (**d-f,k,l**). Source data are provided as a Source Data file.

Supplementary Fig.4

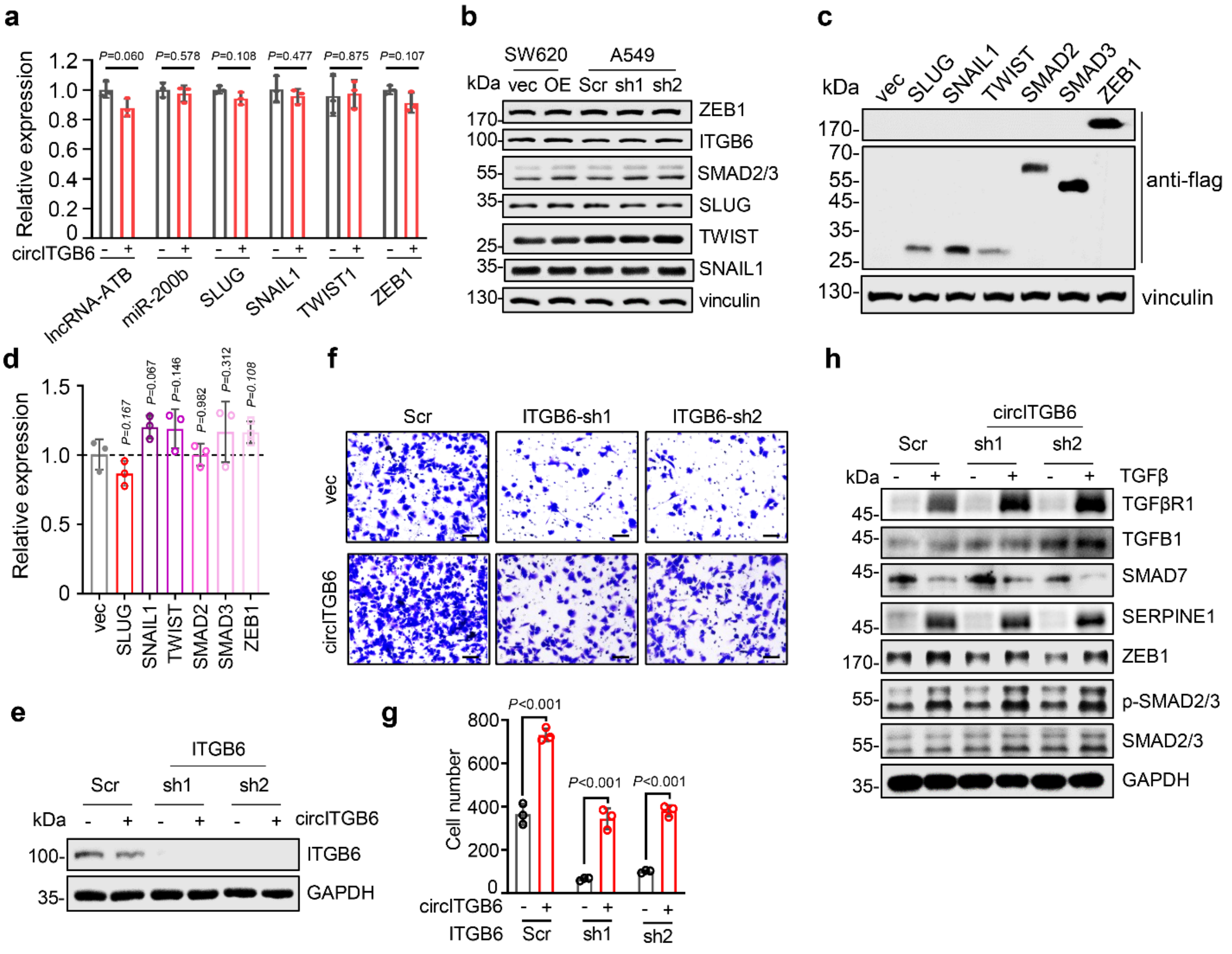

Supplementary Fig. 4 | circITGB6 has no effect on canonical EMT-related TFs or ncRNAs in TGFβ signaling. a qPCR assays for measuring the RNA levels of EMT-related TFs or ncRNAs in SW620 cells with circITGB6 overexpression. **b** Immunoblotting assays show that circITGB6 has little effect on the expression of ITGB6 and EMT-related TFs. **c,d** qPCR assays for measuring the expression of circITGB6 (**d**) in A549 cells with indicated EMT-related TFs overexpression (**c**). **e-g** Transwell migration assays (**f**) and the numbers of migrated cells (**g**) in circITGB6-overexpressing stable A549 cells with or without ITGB6 knockdown (**e**). Scale bar,20 μm. **h** Immunoblotting assays for the expressions of TGFβR1, TGFB1, SMAD7 and SERPINE1 in A549 cell with circITGB6 knockdown upon TGFβ stimulation. Data represent mean ± sd from three independent experiments (**a**,**d**,**g**). Significance of differences was determined by unpaired, two-tailed Student's *t*-test (**a**,**d**,**g**). Source data are provided as a Source Data file.

Supplementary Fig.5

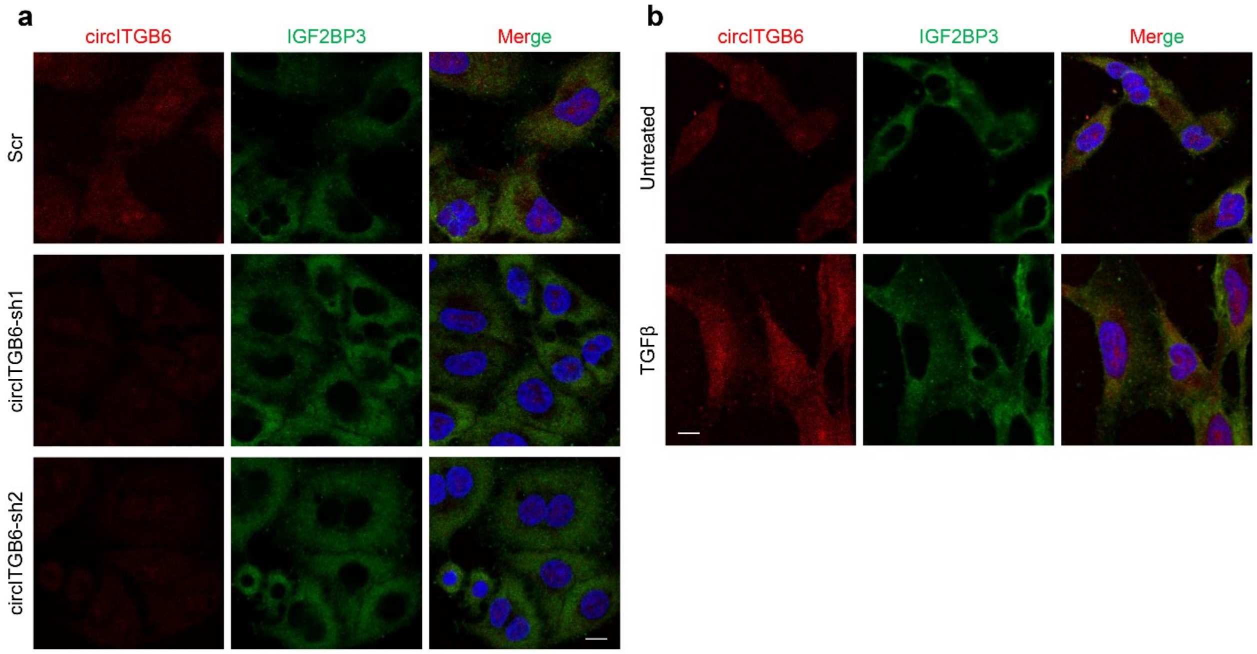

Supplementary Fig. 5 | circITGB6 colocalizes with IGF2BP3. a,**b** Representative images of the co-localization between circITGB6 (red) and IGF2BP3 (green) in A549 cell with circITGB6 depletion **(a)** or with 5 ng/mL TGFβ treatment for 72 hours **(b)**. Scale bar: 20 µm.

Supplementary Fig.6

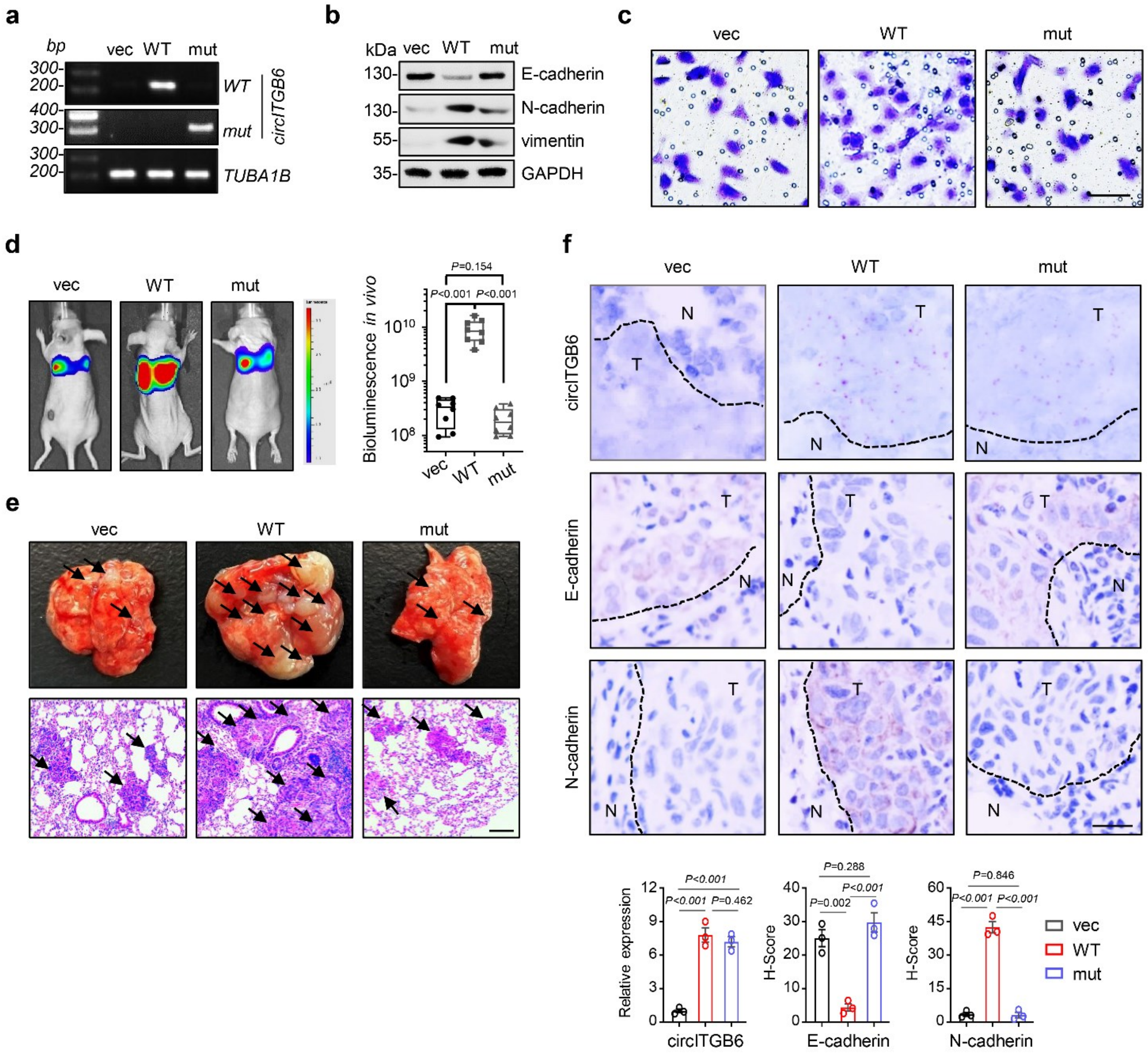

Supplementary Fig. 6 | circITGB6-IGF2BP3 interaction is essential for tumor metastasis. a Identification of A549 stable cells overexpressing circITGB6 wild-type (WT) or IGF2BP3-binding deficient mutant (mut) by RT-PCR assays using indicated primers. **b,c** Immunoblot assays of EMT markers (**b**) and Transwell migration assays (**c**) in different A549 stable cells. Scale bar: 20 μm. **d** Representative bioluminescence imaging of tumor metastasis (**left**) and quantitation of bioluminescence (**right**). The horizontal lines represent the median; the bottom and top of the boxes represent the 25th and 75th percentiles, respectively; and the vertical bars represent the range of the data. **e** Representative images for H&E staining of mouse lung tissues. The metastatic nodules indicated by the black arrows. Scale bar: 100 μm. **f** Representative images and quantitation analysis for the foci of circITGB6 (red point) or IHC staining of E-cadherin and N-cadherin in metastatic lesions in the lung from mice tail-vein injected with A549 stable cells. Scale bar: 20 μm. Data represent mean ± s.e.m (**d, f**). Significance of differences was determined by one-way ANOVA test (**d, f**). Source data are provided as a Source Data file.

Supplementary Fig.7

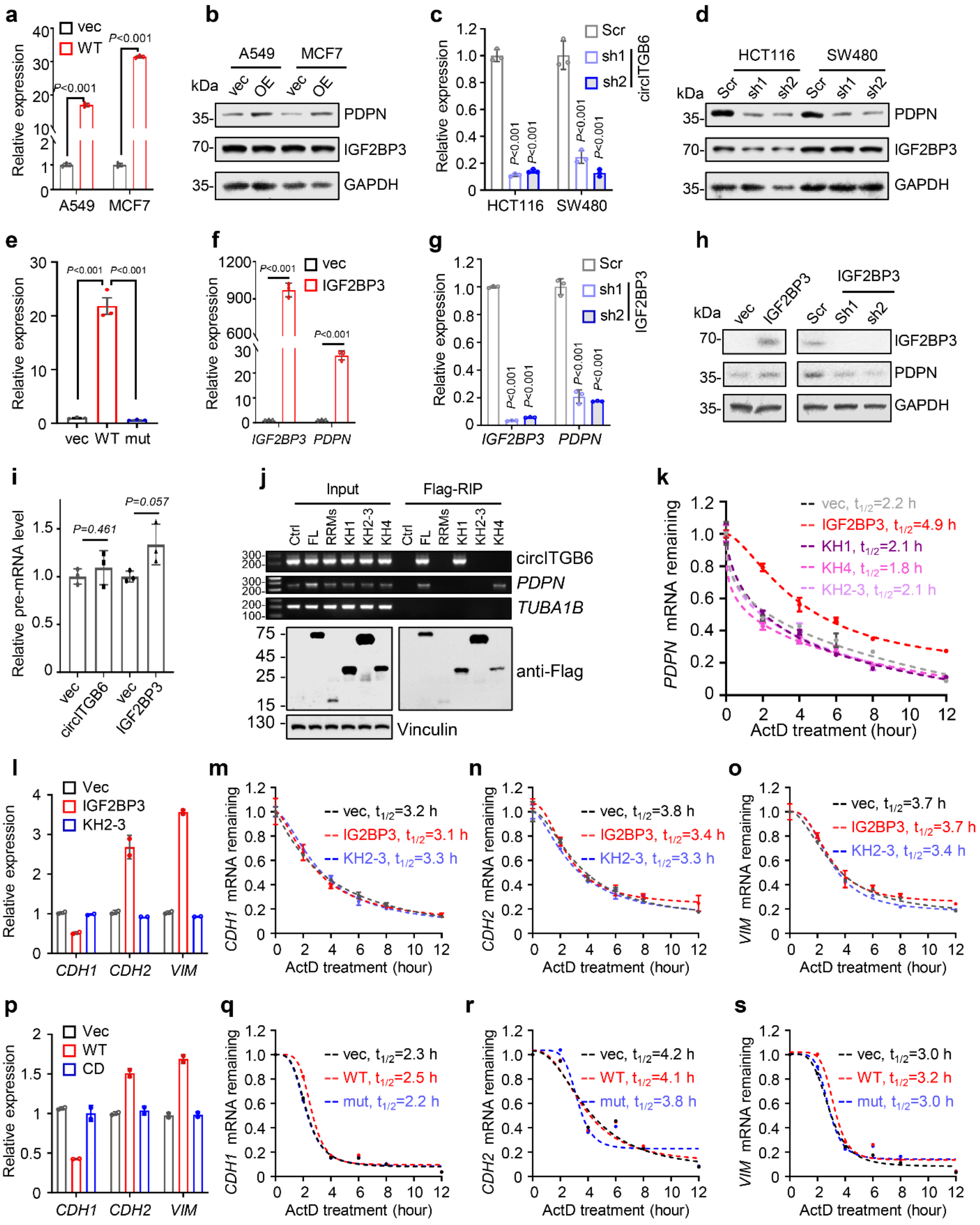

Supplementary Fig. 7 |circITGB6 enhances *PDPN* mRNA stability dependent on its association with IGF2BP3. a-d qPCR assays (**a**,**c**) and western blotting (**b**,**d**) to examine the effects on *PDPN* mRNA and protein levels in different cancer cells with circITGB6 overexpression (OE) (**a**,**b**) or knockdown (**c**,**d**). **e** qPCR assays for the expression of *PDPN* mRNA in A549 cells with circITGB6 wild type (WT) or IGF2BP3 interaction-deficient mutant (mut) overexpression. **f-h** qPCR assays (**f**,**g**) and western blotting (**h**) to examine the effects on *PDPN* mRNA and protein levels in A549 cells with IGF2BP3 overexpression (**f,h**) or knockdown (**g,h**). **i** qPCR assays of *PDPN* pre-mRNA in A549 cells with circITGB6 or IGF2BP3 overexpression. **j** RT-PCR assays for the enrichment of circITGB6 or *PDPN* mRNA in RIP experiments with anti-Flag agarose in A549 cells stably expressing flag-tagged full-length (FL) or truncated IGF2BP3 mutants (indicated by schematic diagrams in Fig. 6f, respectively). **k** Measurement of the half-life of *PDPN* mRNA levels in A549 cells stably expressing IGF2BP3 or the truncated mutants after actinomycin D (ActD) treatment for indicated times. **l-o** Measurement of the expressions and half-lives of *CDH1, CDH2 and VIM* mRNA levels in A549 cells with stable expression of IGF2BP3 or KH2-3 mutant. **p-s** Measurement of the expressions and half-lives of *CDH1, CDH2 and VIM* mRNA levels in A549 cells with circITGB6 wild type (WT) or IGF2BP3 interaction-deficient mutant (mut) overexpression. Data represent mean ± sd from three independent experiments (**a**,**c**,**e-g,i,k-s**). Significance of differences was determined by two-tailed Student's *t*-test (**a**,**c**,**e-g,i**). Source data are provided as a Source Data file.

Supplementary Fig.8

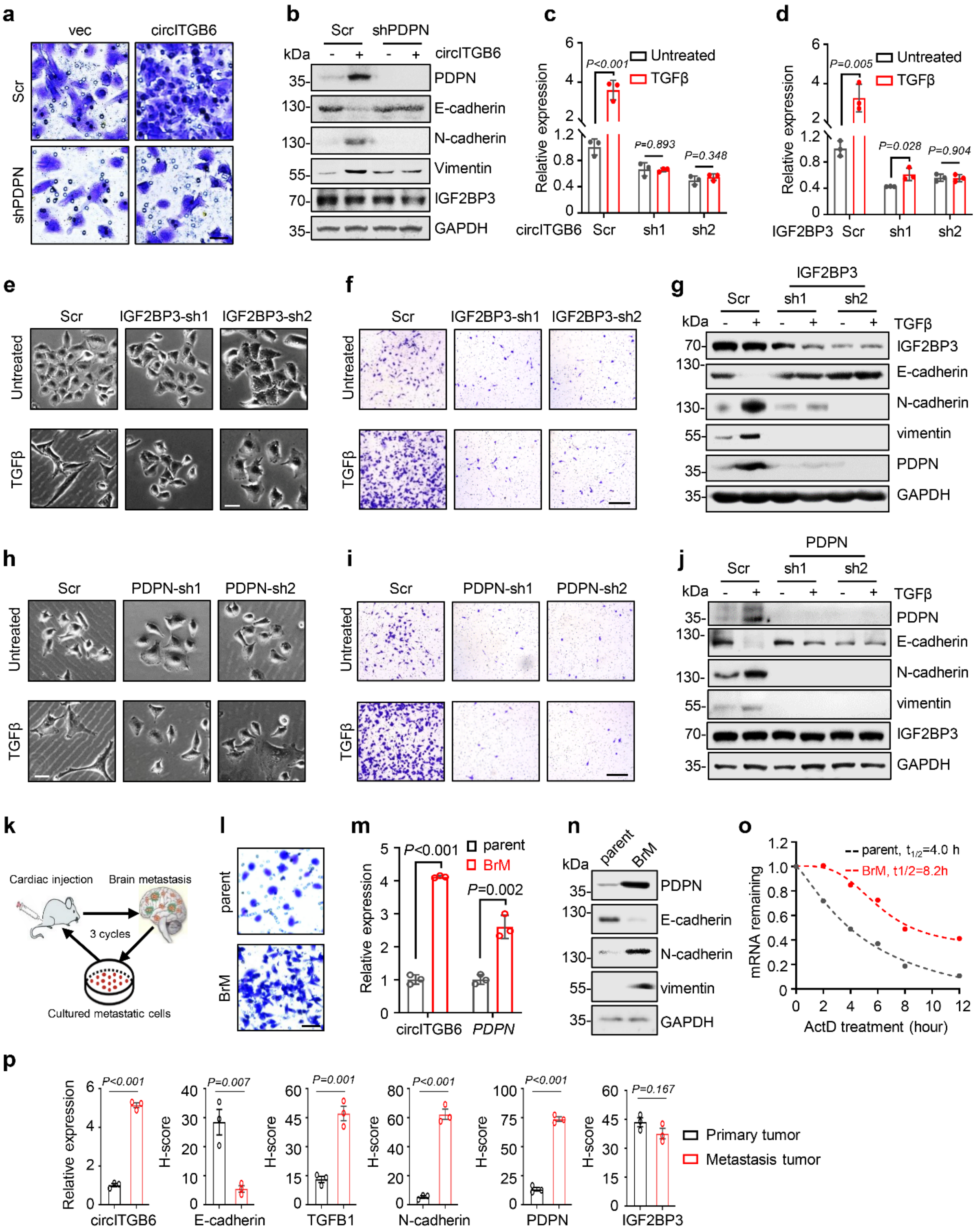

a
vec
circITGB6
Scr
shPDPN
b
Scr
shPDPN
kDa
circITGB6
PDPN
E-cadherin
N-cadherin
Vimentin
IGF2BP3
GAPDH
c
Relative expression
P<0.001
P=0.893
P=0.348
Untreated
TGFβ
circITGB6
Scr
sh1
sh2
d
P=0.005
P=0.028
P=0.904
IGF2BP3
e
IGF2BP3-sh1
IGF2BP3-sh2
Untreated
TGFβ
f
g
vimentin
h
PDPN-sh1
PDPN-sh2
i
j
k
Cardiac injection
Brain metastasis
3 cycles
Cultured metastatic cells
l
parent
BrM
m
P<0.001
P=0.002
PDPN
n
o
parent, t1/2=4.0 h
BrM, t1/2=8.2h
mRNA remaining
ActD treatment (hour)
p
P=0.007
P=0.001
P=0.167
H-score
E-cadherin
TGFB1
N-cadherin
Primary tumor
Metastasis tumor

Supplementary Fig. 8 | circITGB6/IGF2BP3 induced EMT process and tumor metastasis dependent on PDPN. a,b Transwell migration assays (**a**) and immunoblot analysis of PDPN, IGF2BP3 and EMT markers (**b**) for stably circITGB6-overexpression A549 cells with or without PDPN knockdown. **c** qPCR assays for *PDPN* mRNA levels in A549 cell with circITGB6 knockdown upon TGFβ stimulation. **d-g** qPCR assays for *PDPN* mRNA levels(**d**), representative images of cell morphology (**e**), transwell migration assays (**f**)and immunoblotting assays for EMT markers, PDPN and IGF2BP3 (**g**) in A549 cell with circITGB6 knockdown upon TGFβ stimulation. Each dot represents a technical replicate of the assays(**d**). **h-j** Representative images of cell morphology (**h**), transwell migration assays (**i**)and immunoblotting assays for EMT markers, PDPN and IGF2BP3 (**j**) in A549 cell with circITGB6 knockdown upon TGFβ stimulation. **k** Schematic diagram shows the selection of highly brain-metastatic A549 cells. A549 cells (2×10^5) were intracardially injected into immunodeficient nude mice. 26~30 days later, the brain-metastasized cancer cells were isolated and cultured *in vitro* for ~15 days. After three-round training, the highly brain-metastatic A549 cells were obtained and named as BrM. **l-o** Transwell migration assays (**l**), qPCR assays for measuring circITGB6 and PDPN expression (**m**), immunoblot assays of EMT markers and PDPN expression (**n**) and measurement of *PDPN* mRNA half-life (**o**) in A549 parent cells and the BrM derivates. Scale bar: 20 μm. **p** Quantitation analysis of TGFB1, E-cadherin, N-cadherin, PDPN and IGF2BP3 IHC staining and the foci of circITGB6 (red point) in the primary tumors and metastatic lesions (Related to Fig.6j). Data represent mean ± sd from three independent experiments (**c**,**d**,**m**) ; Data represent mean ± s.e.m (**p**). Significance of differences was determined by two-tailed Student's *t*-test (**c**,**d**,**m,p**). Source data are provided as a Source Data file.

Supplementary Fig.9

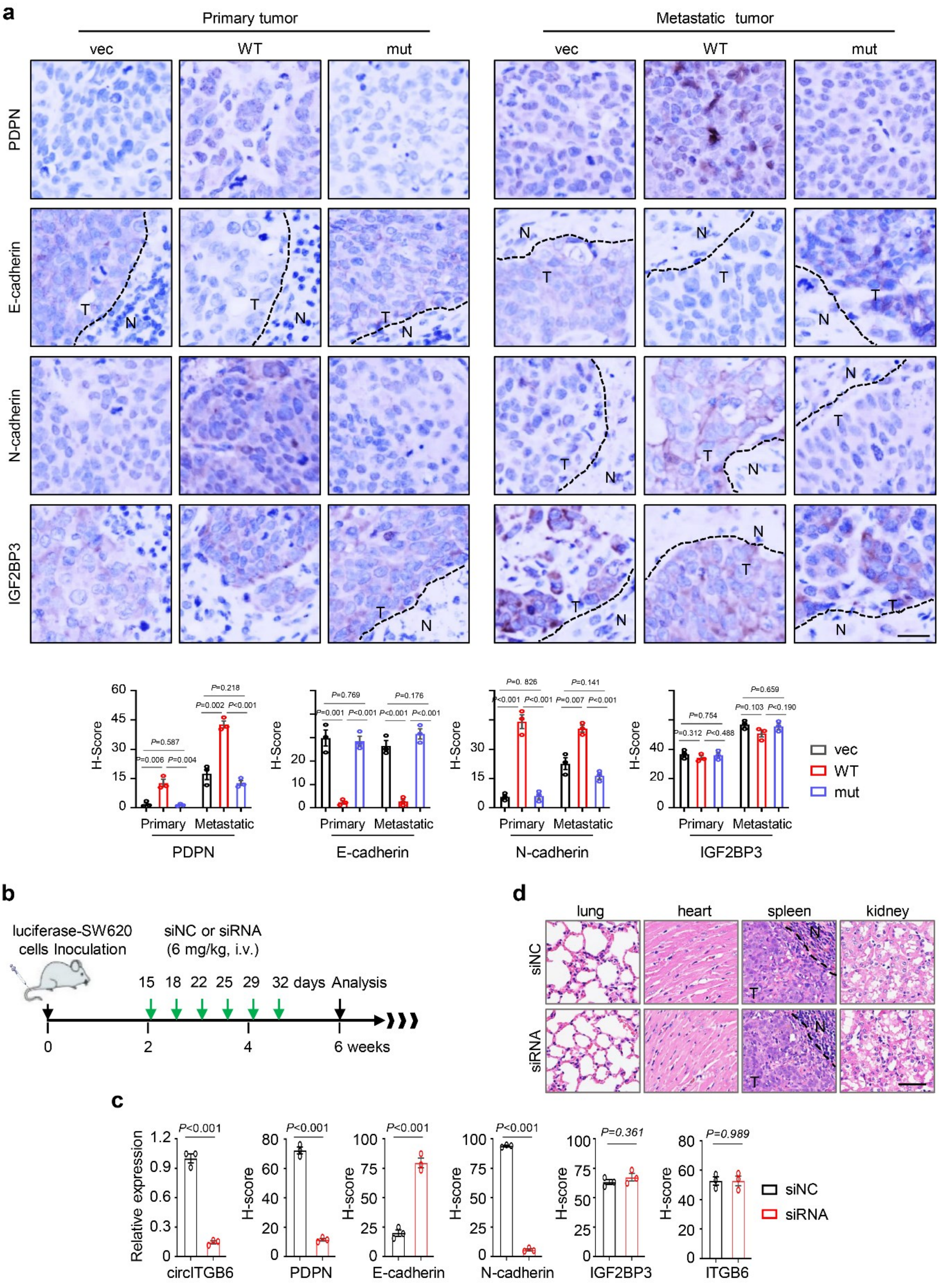

Supplementary Fig. 9 | siRNA targets circITGB6 effectively suppresses liver metastasis. a Representative images and quantitation analysis for IHC staining of PDPN, E-cadherin, N-cadherin and IGF2BP3 in the primary tumors and

metastatic lesions within the liver from mice intrasplenic injection of SW620 cells stably expressing the empty vector, or indicated circITGB6 variants. **b-d** Schematic diagrams of experimental design and treatment schedules. Mice were injected with Eca109 or SW620 cells stably expressing luciferase (2×10^6 for SW620 cells) to establish liver metastasis. Two weeks later, mice were randomly divided into two groups and intravenously injected twice a week for additional three weeks with PEI-coated siNC or siRNA (6 mg / kg body weight) (**b**). n = 7 mice per group. Quantitation analysis of the foci of circITGB6 (red point) and the IHC staining of PDPN, E-cadherin, N-cadherin, IGF2BP3 and ITGB6 in metastatic lesions (**c**). Representative images of H&E staining for main organs in mice injected with SW620 cells (**d**). Scale bar: 20 μm (**a,d**). Data represent mean ± s.e.m (**a,c**). Significance of differences was determined by one-way ANOVA test (**a**), two-tailed Student's *t*-test (**c**). Source data are provided as a Source Data file.

Supplementary table 1. Fold changes of circRNAs in MCF10A, A549, HCT116 and HT29 cells with TGFβ treatment

Cell lines		MCF10A		A549		HCT116		HT29	
circRNA(hsa_circ_)		Fold changes	*P* value	Fold changes	*P* value	Fold changes	*P* value	Fold changes	*P* value
	**0000231**	0.566	<0.001	0.701	<0.001	0.714	0.027	0.133	0.002
	**0001006**	0.396	0.006	0.036	0.009	0.136	<0.001	0.202	0.004
	**0001573**	0.310	0.001	0.572	0.039	0.568	<0.001	1.015	0.890
	**0069982**	0.351	0.003	0.119	0.004	0.516	<0.001	0.157	<0.001
	**0000566**	0.501	<0.001	0.634	<0.001	0.435	<0.001	0.886	0.180
	**0031607**	0.211	0.002	0.320	0.010	ND	ND	ND	ND
**Downregulated circRNAs**	**0006633**	0.942	0.475	0.522	0.047	0.503	0.107	0.495	0.006
	**0001355**	0.602	<0.001	0.665	0.006	0.561	<0.001	0.925	0.428
	**0025768**	ND	ND	ND	ND	ND	ND	ND	ND
	**0139171**	ND	ND	0.455	0.004	0.219	0.009	0.781	0.225
	**0001460**	0.003	0.002	0.001	0.009	0.515	<0.001	0.333	0.049
	**0008359**	0.642	0.037	0.470	0.024	0.981	0.751	0.117	0.018
	**0002928**	0.646	0.001	1.035	0.741	ND	ND	ND	ND
	**0002220**	0.801	0.028	1.063	0.705	1.759	<0.001	0.996	0.930
	**0056856**	24.281	<0.001	22.576	<0.001	33.723	<0.001	62.328	0.001
**Upregulated circRNAs**	**0001610**	0.648	<0.001	0.715	0.005	1.298	0.003	0.979	0.620
	**0000972**	2.681	<0.001	2.859	<0.001	9.530	0.001	3.186	<0.001
	**0009043**	0.801	0.248	1.194	0.133	1.463	0.014	1.194	0.314

qPCR assays to measure the expression of dysregulated circRNAs identified in RNA-seq data. Fold changes were normalized to the untreated samples for each experiment (set as a value of 1.0). Data represent mean from three independent experiments. ND, not detected. Significance of differences was determined by unpaired, two-tailed Student's *t*-test.

Supplementary Table 2.Correlation between circITGB6 expression and the clinicopathological features of CRC.

Characteristic		Number of cases	circITGB6 expression		Chi-square Test *P*-value
			Low，n (%)	High，n (%)	
**Age, yr**	< 60	28	11(39.3)	17(60.7)	0.1835
	≥ 60	52	13(25.0)	39(75.0)	
**Gender**	Male	47	15(31.9)	32(68.1)	0.8939
	Female	33	11(33.3)	22(67.7)	
**Tumor location**	Rectum	19	7(36.8)	12(63.2)	0.7441
	colon	61	20(32.8)	41(67.2)	
**Tumor size**	< 5 cm	63	19(30.2)	44(69.8)	0.9524
	≥5 cm	17	5(29.4)	12(70.6)	
**Clinical stage**	I+II	59	21(35.6)	38(64.4)	0.4200
	III	21	5(23.8)	16(76.2)	
**Primary tumor**	T1-T2	9	5(55.6)	4(44.4)	0.1170
	T3-T4	71	21(29.6)	50(70.4)	
**Lymph node status**	No metastasis	45	38(84.4)	7(15.6)	**0.0013**
	Metastasis	35	17(48.6)	18(51.4)	
**Survival**	Alive	36	21(58.3)	15(41.7)	**0.0014**
	Dead	44	10(22.7)	34(77.3)	

Significance of differences (*P*-value) was determined by one-sided Chi-square Test.

Supplementary Table 3.Univariate and multivariate cox regression analysis of prognostic factors in patients of CRC

Variable	Subset	Hazard ratio for DSS (95% CI)	*P* value
**Univariate analysis (n=80)**			
**Age, yr**	< 60 *vs.* ≥ 60	0.9550 (0.474-1.924)	0.5550
**Gender**	Male *vs.* Female	0.8009 (0.389-1.648)	0.5464
**Tumor location**	Rectum *vs.* Colon	0.5587 (0.253-1.234)	0.1497
**Tumor size**	< 5 cm *vs.*≥ 5 cm	0.2435 (0.060-0.994)	**0.0491**
**Clinical stage**	I+II *vs.* III	1.5860 (0.718-3.501)	0.2539
**Primary tumor**	T1-T2 *vs.* T3-T4	6.1610 (1.361-27.89)	**0.0183**
**Lymph node status**	No metastasis *vs.* Metastasis	2.8510 (1.329-6.110)	**0.0066**
**circITGB6 expression**	Low *vs.* High	0.5193 (0.197-1.372)	**0.0098**
**Multivariate analysis (n=80)**			
**Tumor size**	< 5 cm *vs.* ≥ 5 cm	0.900 (0.367-2.211)	0.818
**Primary tumor**	T1-T2 *vs.* T3-T4	1.528 (0.636-3.666)	0.343
**Lymph node status**	No metastasis *vs.* Metastasis	0.781 (0.373-1.639)	**0.041**
**circITGB6 expression**	Low *vs.* High	0.402 (0.145-1.119)	**0.028**

Significance of differences (*P*-value) was determined by Cox regression analysis.

Supplementary Table 4. Identified IGF2BP3 peptides by mass spectrometry

Sequences	DeltaScore	Intensity
AIEALSGK	0.3682	31074484
ALQSGPPQSR	0.6528	2604388
DQTPDENDQVVVK	0.7663	148393712
EENFVSPK	0.5882	3596327
EEVKLEAHIR	0.6807	63343800
FTEEIPLK	0.7207	153325632
IAPAEAPDAK	0.64	806459
IELHGKPIEVEHSVPK	0.6178	130677144
IKEENFVSPK	0.5902	96209664
IKEENFVSPKEEVK	0.6552	11726721
ILAHNNFVGR	0.5658	17142562
IPVSGPFLVK	0.7156	184306528
IQEILTQVK	0.2367	53760672
ITGHFYACQVAQR	0.7187	36978540
ITISPLQELTLYNPER	0.7213	15463082
KIQEILTQVK	0.5274	51107060
LLVPTQFVGAIIGK	0	973572
LNGFQLENFTLK	0.7547	8250078
MVIITGPPEAQFK	0.7702	282413
KIQEILTQVK	0.5596	34942032
QKPCDLPLR	0.5671	15877153
KIQEILTQVK	0.5647	5902263
SILEIMHK	0.5336	22603280
SITILSTPEGTSAACK	0.8473	32532712
TGYAFVDCPDESWALK	0.8521	924496
TVNELQNLSSAEVVVPR	0.7947	20218102
VAYIPDEMAAQQNPLQQPR	0.781	17900834
VPSFAAGR	0.7368	127081720
TGYAFVDCPDESWALK	0.8521	924496

Supplementary Table 5. List of primers, probes and shRNA sequences

Primers used in qPCR assays		
Name	Sequence (5'-3')	
	Forward primer	Reverse primer
circITGB6	GCGACCATCAGTGAAGAAGAAGGTA	AGCACTCCATCTTCAGAGACGCA
ITGB6	TGCTTATTGGACCTCAGTGTG	CTACTTGGGAGACAGGGTTTTC
IGF2BP3	GTTTATCCCAGCTCTATCAGTCG	TCACCATCCTCACTTTAGCATC
Actin	AGGCCAACCGCGAGAAGATG	GCCAGAGGCGTACAGGGATA
U6	CGCTTCGGCAGCACATATAC	AGGGGCCATGCTAATCTTCT
MMP9	GCCACTACTGTGCCTTTGAGTC	CCCTCAGAGAATCGCCAGTACT
CD44s	CATCCCAGACGAAGACAGTC	GAATGTGTCTTGGTCTCTGGT
CD164	AACGTGACGACTTTAGCGCCCA	ACGCAGCTGTTTCGACCTTCAC
TWIST1	GCCAGGTACATCGACTTCCTCT	TCCATCCTCCAGACCGAGAAGG
circGNB1	AGTGGGAAGAATCCAAATGC	GGCTTCTGGTCTGGTCTCC
circSMAD2	TGAAGATGGAGAAACAAGTGACC	TCCCACTGATCTATCGTATTTGG
hsa_circ_0002220	GCCATTCATCAAAGAGGGCCTGGA	GCTTATGCAAGATAACGGGTCCAG
hsa_circ_0000972	ATTCTGCTGATTTTTCAGGGTCAAC	ATAAAGGCCCAAAAGGGTAGCAAC
hsa_circ_0009043	GAGAGACTGCCATGAAGCAAAATC	CTCTAGGACTGGAAGACACAGCATT
hsa_circ_0001573	GCATCTGCGGAAAGTCACTG	ACAGTCGGAGCAACCTGTGC
hsa_circ_0001460	GCTGCAACCCTGGATATTCTAACA	CTTCACTCTTGAGCACTGAATTTCA
hsa_circ_0008359	ATATGGCCAACTTGATTGCAGC	CCATATACAAGGCCCCATTAGCA
hsa_circ_0069982	ATGCCTATTGCCATGCTTTTACACA	CCTGATATAATAATGGCTTCATGTCTGC
hsa_circ_0001610	GGTGCTTGTGGTGATTGTGGTG	TGCTAAGGAATCCAAGCATGGCC
hsa_circ_0002928	TCATGTCATTGATCGCTGGCTTG	ATTCAGCTCCTGTAACATGGCCTTG
mouse circItgb6	CCCAAGAAGAGTGTGCTGAC	CACAGCACTCCATCCTCAGA
ZEB1	ACAACAACAAGACACTGCTGT	TGGACAGGTGAGTAATTGTGAA
lncRNA-ATB	TCTGGCTGAGGCTGGTTGAC	ATCTCTGGGTGCTGGTGAAGG
SNAIL1	TGCCCTCAAGATGCACATCCGA	GGGACAGGAGAAGGGCTTCTC
TWIST1	GCCAGGTACATCGACTTCCTCT	TCCATCCTCCAGACCGAGAAGG
SLUG	ATCTGCGGCAAGGCGTTTTCCA	GAGCCCTCAGATTTGACCTGTC
CDH1	CCACCAAAGTCACGCTGAAT	CCAGGAGAGGAGTTGGGAAA
CDH2	TTGCTGTTTTGGACCGAGAA	AGCGTTCCTGTTCCACTCAT
VIM	TGTTTCCAAGCCTGACCTCA	CGGTACTCAGTGGACTCCTG
PDPN	GTGCCGAAGATGATGTGGTGAC	GGACTGTGCTTTCTGAAGTTGGC
pre-mRNA *ITGB6*	AAGAGAAAAGATGCCCCTCAC	CAGTTGCAGTACTCGCCAG
pre-mRNA *PDPN*	ACTACAGGTTTGGAAGGCGG	TCTTCCCAGGACACTGACCA

Primers used in RT-PCR assays		
Name	Sequence (5'-3')	
	Forward primer	Reverse primer

PDPN	GTGCCGAAGATGATGTGGTGAC	GGACTGTGCTTTCTGAAGTTGGC
TUBA1B	GCCCTACAACTCCATCCTCA	GTCAACATTCAGGGCTCCAT
CD44	AATGCCTTTGATGGACCAATTAC	GCTCACGTCATCATCAGTAGG
circITGB6-WT	GTGGTGACCCCTGTAACTCTAAACG	AGCACTCCATCTTCAGAGACGCA
circITGB6-mut	ACAGGGGTCACCACAGCATCC	ACAGGGGTCACCACAGGTAG
linear transcript	GTTTTAGGGCGTTAGAGTAGGC	AGCACTCCATCTTCAGAGACGCA
circITGB6 divergent	GTGGTGACCCCTGTAACTCTAAACG	AGCACTCCATCTTCAGAGACGCA
circITGB6 convergent	GTAACGGCGACTGTGACTGTGGTG	ACAGGGGTCACCACAGGTAG
circItgb6 divergent	CCCAAGAAGAGTGTGCTGAC<br>GGCGACCCCTGTAACTCTAA	CACAGCACTCCATCCTCAGA

Primers for *in vitro* transcription

Name	Sequence (5'-3')
T7-circITGB6 WT and mut-sense	TAATACGACTCACTATAGGGGACTGTGTTTGTGGCAA
sense DNA splint	CACTTGCCACAAACACAGTCCCCGCGCCCGCTGCAGAGCACTCCAT
T7-circITGB6 WT and mut-antisense	TAATACGACTCACTATAGGGTTTGTGCAAACACACTT
antisense DNA splint	GGCAAGTGTGTTTGCACAAACCCTGGAGCCTCAGGACCAACCTGTG

Biotin- or Cy3- labeled DNA or RNA probes

Name	Sequence (5'-3')
antisense circITGB6 for RNA pull-down	CACAGTCGCCGTTACCTTCTTCTTCACTGA
sense circITGB6 for RNA pull-down	TCAGTGAAGAAGAAGGTAACGGCGACTGTG
circITGB6 FISH probe	CAGTCACAGTCGCCGTTACCTTCTTCTTCACTGATGGT
U6 FISH probe	GCTAATCTTCTCTGTATCGTTCCAATTTTAGTATATGTGCTGCCG
18S rRNA FISH probe	CATGGCTTAATCTTTGAGAC
circITGB6 northern blotting probe	CAGTCACAGTCGCCGTTACCTTCTTCTTCACTGATGGT
Actin northern blotting probe	ATGATCTGGGTCATCTTCTCGCGGTTGGCC
circITGB6/ITGB6 northern blotting probe	ACTCAATGCAGCTCCGTTTAGAGTTACAGGGGTCAC
M1 probe	ACUCUAAACGGAGCUGCAUUGAGUGCCACC
M1-mut probe	ACUCUAAACGGAGCUGGUAAGAGUGCCACC
M2 probe	ACUAGCUGGUGCGACCAUCAGUGAAGAAGA
control RNA probes	UUGUACUACACAAAAGUACUG
PDPN 3'UTR RNA pull-down	AGGGACAGGGCACAGAGTCAGAAACGGTCT
PDPN 3'UTR RNA pull-down	GGGGCAGGTGGGAGCAAGCTTAGAGTCACC

shRNA sequences

Name	Sequence (5'-3')
circITGB6-sh1	ATCAGTGAAGAAGAAGGTAAC
circITGB6-sh2	AGAAGAAGGTAACGGCGACTG
IGF2BP3-sh1	CGGTGAATGAACTTCAGAATT
IGF2BP3-sh2	GCAGGAATTGACGCTGTATAA
PDPN-sh1	GCTATAAGTCTGGCTTGACAA
PDPN-sh2	CGGCTTCATTGGTGCAATCAT

ITGB6-sh1	GAGGAATACTAACTCTGTTGCATTA
ITGB6-sh2	CCATTTCACTCAGTTGCACAGTCTT
QKI-sh1	CCGAAGCTGGTTTAATCTATA
QKI-sh2	CTGATGCTGTGGGACCTATTG

Primers for plasmid construction

Name	Sequence (5'-3')	
	Forward primer	Reverse primer
circITGB6 WT and mut	GTAACGGCGACTGTGACT	CTTCTTCTTCACTGATGGTCG
IGF2BP3 FL	ATGAACAAACTGTATATCGGA	TTACTTCCGTCTTGACTGAG
IGF2BP3 RRMs	ATGAACAAACTGTATATCGGA	TCACATGGTTTCTGCTTGG
IGF2BP3 KH1	ATGAACAAACTGTATATCGGA	AATAGACTTACAAGCCG
IGF2BP3 KH2-3	GAGATCCCCTTGAAGATTTTA	AATTCTTCCCTGAGCCT
IGF2BP3 KH4	GAAGAGGTGAAACTTGAAGCTCA	TTACTTCCGTCTTGACTGAG
SLUG	ATGCCGCGCTCCTTCCTGGT	TCAGTGTGCTACACAGCAGC
SNIAL1	ATGCCGCGCTCTTTCCTCG	TCAGCGGGGACATCCTGAGC
TWIST	ATGATGCAGGACGTGTCCAG	CTAGTGGGACGCGGACATG
SMAD2	ATGTCGTCCATCTTGCCATTC	TTATGACATGCTTGAGCAAC
SMAD3	ATGTCGTCCATCCTGCCTT	CTAAGACACACTGGAACAGC
ZEB1	ATGGCGGATGGCCCCAGGT	TTAGGCTTCATTTGTCTTTTC
QKI	ATGGTCGGGGAAATGGAAACG	GTTGCCGGTGGCGGCTC
