# Supplementary material for: A circular RNA activated by TGFβ promotes tumor metastasis through enhancing IGF2BP3-mediated PDPN mRNA stability
Source: Nat Commun. 2023 Oct 28;14:6876. doi: 10.1038/s41467-023-42571-1 (PMC10613289; doi:10.1038/s41467-023-42571-1)
Supplement: Supplementary file 2 — Reporting Summary [file 41467_2023_42571_MOESM2_ESM.pdf]

nature portfolio

Corresponding author(s): Yong Peng
Last updated by author(s): Jun 25, 2023

Reporting Summary

Nature Portfolio wishes to improve the reproducibility of the work that we publish. This form provides structure for consistency and transparency in reporting. For further information on Nature Portfolio policies, see our Editorial Policies and the Editorial Policy Checklist.

Statistics

For all statistical analyses, confirm that the following items are present in the figure legend, table legend, main text, or Methods section.

n/a	Confirmed	
☐	☒	The exact sample size (*n*) for each experimental group/condition, given as a discrete number and unit of measurement
☐	☒	A statement on whether measurements were taken from distinct samples or whether the same sample was measured repeatedly
☐	☒	The statistical test(s) used AND whether they are one- or two-sided<br>*Only common tests should be described solely by name; describe more complex techniques in the Methods section.*
☒	☐	A description of all covariates tested
☒	☐	A description of any assumptions or corrections, such as tests of normality and adjustment for multiple comparisons
☐	☒	A full description of the statistical parameters including central tendency (e.g. means) or other basic estimates (e.g. regression coefficient) AND variation (e.g. standard deviation) or associated estimates of uncertainty (e.g. confidence intervals)
☐	☒	For null hypothesis testing, the test statistic (e.g. *F*, *t*, *r*) with confidence intervals, effect sizes, degrees of freedom and *P* value noted<br>*Give P values as exact values whenever suitable.*
☒	☐	For Bayesian analysis, information on the choice of priors and Markov chain Monte Carlo settings
☒	☐	For hierarchical and complex designs, identification of the appropriate level for tests and full reporting of outcomes
☐	☒	Estimates of effect sizes (e.g. Cohen's *d*, Pearson's *r*), indicating how they were calculated

Our web collection on statistics for biologists contains articles on many of the points above.

Software and code

Policy information about availability of computer code

Data collection	qPCR raw data were collected by QuantStudio 6 Operating Software v1.6.1. For Mass spectrometry analysis, protein identification and quantification were accomplished by Proteome Discoverer software v1.4. Bioluminescence in vivo was collected by IVIS Spectrum Operating Software Livingmage v3.2. Western blot images were collected by Bio-Rad Image Lab Software v6. IF and RNA FISH images were collected by Zeiss Zen Lite v3.1. RNAscope ISH, IHC and H&E staining images were collected by Akoya Biosciences inform software v2.4.2.
Data analysis	IHC staining scoring was quantified with Akoya Biosciences inform software v2.4.2. Relative intensity was quantified with Image J software v1.8.0. All statistical analyses were carried out using GraphPad Prism v9.0 and SPSS v26.0.

For manuscripts utilizing custom algorithms or software that are central to the research but not yet described in published literature, software must be made available to editors and reviewers. We strongly encourage code deposition in a community repository (e.g. GitHub). See the Nature Portfolio guidelines for submitting code & software for further information.

Data

Policy information about availability of data

All manuscripts must include a data availability statement. This statement should provide the following information, where applicable:

- Accession codes, unique identifiers, or web links for publicly available datasets
- A description of any restrictions on data availability
- For clinical datasets or third party data, please ensure that the statement adheres to our policy

- circRNA sequencing data were deposited in the Gene Expression Omnibus under accession number: GSE165576.
- circBase, a database for merged and unified data sets of human circRNAs.
(http://www.circbase.org/)
- RBPmap, a database for Motifs Analysis and Prediction of RNA binding proteins.
(http://rbpmap.technion.ac.il/)
- TransCirc, a database that provide comprehensive evidences supporting the translation potential of circular RNAs.
(https://www.biosino.org/transcirc/)
- riboCIRC, a database for of translatable circRNAs
(http://ribocirc.com/index.html)

Human research participants

Policy information about studies involving human research participants and Sex and Gender in Research.

Reporting on sex and gender	Sex and gender were identified as insignificant factor in prognosis analysis. Source data disaggregated for sex has been collected and provided within the Supplementary table 2.
Population characteristics	80 human colon cancer tissues (47 males and 33 females, aged from 33 to 85) were used. No personal information about the identity of the patients was made accessible to the researchers.
Recruitment	80 human colon cancer tissues were collected with patient consent from the biobank of Shanghai Outdo Biotech Co., Ltd. No self-selection bias was anticpated.
Ethics oversight	The study was approved by the Ethics Committee of Shanghai Outdo Biotech Co., Ltd. (2005DKA21300)

Note that full information on the approval of the study protocol must also be provided in the manuscript.

Field-specific reporting

Please select the one below that is the best fit for your research. If you are not sure, read the appropriate sections before making your selection.

☒ Life sciences ☐ Behavioural & social sciences ☐ Ecological, evolutionary & environmental sciences

For a reference copy of the document with all sections, see nature.com/documents/nr-reporting-summary-flat.pdf

Life sciences study design

All studies must disclose on these points even when the disclosure is negative.

Sample size	For animal experiments, 5-8 mice were sacrificed per experiments group. Specific sample size for each experiments is indicated in the manuscript. The sample size was determined based on previous experience with metastasis model of A549 (6 weeks after tail-vein injection), Capan-2(8 weeks after intracardiac injection), and SW620 (8 weeks after intraspelenic injection) cell lines. For clinical data, sample size was based on our previous experience and the number of samples with available prognostic information.<br>For cell and biochemical data, the sample size was based on our previous experience and we aimed to collect data from three biological replicated when possible.
Data exclusions	No data have been excluded from the analysis.
Replication	All attempts for replication were successful by different co-authors of this study. For in vitro experiments, at least three biologically independent experiments were performed for all experiments unless otherwise stated. For in vivo experiments, n=5-8 mice/group mice were used.
Randomization	All mice and cells were allocated in random. No bias is sample allocation was involved.
Blinding	Investigators were blinded to group allocation during data collection and analysis.

Reporting for specific materials, systems and methods

We require information from authors about some types of materials, experimental systems and methods used in many studies. Here, indicate whether each material, system or method listed is relevant to your study. If you are not sure if a list item applies to your research, read the appropriate section before selecting a response.

Materials & experimental systems

n/a	Involved in the study
☐	☒ Antibodies
☐	☒ Eukaryotic cell lines
☒	☐ Palaeontology and archaeology
☐	☒ Animals and other organisms
☒	☐ Clinical data
☒	☐ Dual use research of concern

Methods

n/a	Involved in the study
☒	☐ ChIP-seq
☒	☐ Flow cytometry
☒	☐ MRI-based neuroimaging

Antibodies

Antibodies used

anti-IGF2BP3 (Abcam, Cat#ab177477, 1:2000 dilution for WB 1:100 dilution for IHC or IF assays);
anti-TGFβR1(Abcam, Cat#ab235578, 1:1000 dilution)
anti-LaminA/C (Abways, Cat#CY5222, 1:2000 dilution);
anti-SMAD7 (ABclonal, Cat#A12343, 1:1000 dilution);
anti-flag (Abmart, Cat#M20008, 1:5000 dilution);
anti-PDPN (Proteintech, Cat#11629-1-AP, 1:1000 dilution for WB assays, 1:400 dilution for IHC assays);
anti-SRSF1 (HuaBio, Cat#ET7107-70, 1:1000 dilution);
anti-YBX1 (Proteintech, Cat#20339-1-AP, 1:1000 dilution);
anti-QKI(Proteintech, Cat#13169-1-AP, 1:1000 dilution);
anti-ITGB6 (Proteintech, Cat#19695-1-AP, 1:1000 dilution for WB assays, 1:200 dilution for IHC assays);
anti-TGFB1 (Proteintech, Cat#21898-1-AP, 1:1000 dilution for WB assays, 1:400 dilution for IHC assays);
anti-SERPINE1 (Proteintech, Cat#13801-1-AP, 1:1000 dilution);
anti-ZEB1 (Proteintech, Cat#21544-1-AP, 1:2000 dilution);
anti-E-cadherin (Cell signaling, Cat# 3195, 1:1000 dilution for WB assays, 1:400 dilution for IHC assays);
anti-GAPDH (Cell signaling, Cat# 8884, 1:5000 dilution);
anti-N-cadherin (Cell signaling, Cat# 13116, 1:1000 dilution for WB assays, 1:125 dilution for IHC assays);
anti-Vimentin (Cell signaling, Cat# 5741, 1:1000 dilution);
anti-SMAD2/3 (Cell signaling, Cat#8685, 1:1000 dilution);
anti-phospho-SMAD2/3 (Cell signaling, Cat#8828, 1:1000 dilution);
anti-slug (Cell signaling, Cat#9585, 1:1000 dilution);
anti-Snail1 (Cell signaling, Cat#3879, 1:1000 dilution);
anti-Twist1 (Cell signaling, Cat#69366, 1:1000 dilution);
anti-Vinculin (Cell signaling, Cat#4650, 1:2000 dilution)
Normal Rabbit IgG (Cell signaling, Cat# 2729, 5 ug/Sample);
HRP-conjungated secondary anti-mouse IgG (Cell signaling, Cat# 7076, 1:10000 dilution);
HRP-conjungated secondary anti-mouse IgG (Cell signaling, Cat# 7074, 1:10000 dilution);
(AF488) goat anti-rabbit IgG (Bioss Cat#bs-0295G-AF488, 1:300 dilution).

Validation

All commercially available antibodies were validated by vendors. Validation statements are provided on the manufacture's website. We examined primary antibodies according to manuals, and got similar results with validation results on manufacturer's website or relevant citations.

- IGF2BP3 antibody used was validated for WB, IHC and IF assays.
(https://www.abcam.com/products/primary-antibodies/imp3-antibody-epr12021-ab177477.html)
- TGFβR1 antibody used was validated for WB assays.
(https://www.abcam.com/products/primary-antibodies/tgf-beta-receptor-i-antibody-epr20923-13-ab235578.html)
- Vinculin antibody used was validated for WB assays.
(https://www.cellsignal.com/products/primary-antibodies/vinculin-antibody/4650)
- slug antibody used was validated for WB assays.
(https://www.cellsignal.com/products/primary-antibodies/slug-c19g7-rabbit-mab/9585)
- Snail1 antibody used was validated for WB assays.
(https://www.cellsignal.com/products/primary-antibodies/snail-c15d3-rabbit-mab/3879)
- Twist1 antibody used was validated for WB assays.
(https://www.cellsignal.cn/products/primary-antibodies/twist1-e7e2g-rabbit-mab/69366)
- LaminA/C antibody used was validated for WB assays.
(http://www.abways.cn/showproduct.asp?cid=CY5222)
- SMAD7 antibody used was validated for WB assays.

(https://abclonal.com.cn/catalog/A12343)
- flag antibody used was validated for WB and IP assays.
(http://www.ab-mart.com.cn/page.aspx?node=%2059%20&id=%20968)
-Vimentin antibody used was validated for WB assays.
(https://www.cellsignal.com/products/primary-antibodies/vimentin-d21h3-xp-rabbit-mab/5741)
-SMAD2/3 antibody used was validated for WB assays.
(https://www.cellsignal.com/products/primary-antibodies/smad2-3-d7g7-xp-rabbit-mab/8685)
-phospho-SMAD2 antibody used was validated for WB assays.
(https://www.cellsignal.com/products/primary-antibodies/phospho-smad2-ser465-467-smad3-ser423-425-d27f4-rabbit-mab/8828)
- PDPN antibody used was validated for WB and IHC assays.
(https://www.ptgcn.com/products/PDPN,D2-40,M2A-Antibody-11629-1-AP.htm)
- E-catherin antibody used was validated for WB and IHC assays.
(https://www.cellsignal.com/products/primary-antibodies/e-cadherin-24e10-rabbit-mab/3195）
- N-catherin antibody used was validated for WB and IHC assays.
(https://www.cellsignal.com/products/primary-antibodies/n-cadherin-d4r1h-xp-rabbit-mab/13116)
- SRSF1 antibody used was validated for WB assays.
(http://www.huabio.cn/product/SF2-antibody-ET7107-70)
- SERPINE1 antibody used was validated for WB assays.
(https://www.ptgcn.com/products/SERPINE1-Antibody-13801-1-AP.htm)
- ZEB1 antibody used was validated for WB assays.
(https://www.ptgcn.com/products/ZEB1-Antibody-21544-1-AP.htm}
- YBX1 antibody used was validated for WB assays.
(https://www.ptgcn.com/products/YBX1-Antibody-20339-1-AP.htm)
-QKI antibody used was validated for WB assays.
(https://www.ptgcn.com/products/QKI-Antibody-13169-1-AP.htm)
- ITGB6 antibody used was validated for WB and IHC assays.
(https://www.ptgcn.com/Products/Pictures/pdf/19695-1-AP.pdf)
- TGFB1 antibody used was validated for WB and IHC assays.
(https://www.ptgcn.com/products/TGF-beta-1-Antibody-21898-1-AP.htm)

Eukaryotic cell lines

Policy information about cell lines and Sex and Gender in Research

Cell line source(s)	HEK293T(ATCC CRL-3216), MCF7 (ATCC HTB-22), SW480 (ATCC CCL-228), SW620 (ATCC CCL-227), HCT116 (ATCC CCL-247), MCF7 (ATCC HTB-38), Capan-2 (ATCC HTB-80), HCC827(ATCC CRL-2868) and A549(ATCC CCL-185) were purchased from ATCC. (Supplemantary Fig.2c) B16 (TCM2) and 4T1(TCM32) were purchased from CellBank (Shanghai, China). MCF-10A was generously provided by Prof. Zhixiong Xiao (Sichuan University). Py2T cells were isolated from a breast tumor of a FVB/NJGpt-Tg (MMTV-PyMT)/Gpt female mouse (GemPharmatech, Strain NO.T004993).
Authentication	Cells were authenticated by STR profiling.
Mycoplasma contamination	All cell line tested negative for mycolasma contamination.
Commonly misidentified lines (See ICLAC register)	The study did not involve commonly misidentified lines.

Animals and other research organisms

Policy information about studies involving animals; ARRIVE guidelines recommended for reporting animal research, and Sex and Gender in Research

Laboratory animals	4~6-week-old female nude mice were purchased from Beijing HFK Bioscience. Mice were housed under 12 light/12 dark cycle,10–15 fresh air exchanges hourly, temperatures of 22±1 °C with 50±10% humidity.
Wild animals	The study did not involve wild animals.
Reporting on sex	Sex has no been reported as a critical factor for tumor metastasis. Female mice were used in this study to establish metastasis model. for their tractability.
Field-collected samples	The study did not involve samples collected from the field.
Ethics oversight	All mouse procedures were approved by the Institutional Animal Care and Use of West China Hospital, Sichuan University.

Note that full information on the approval of the study protocol must also be provided in the manuscript.
